# The Immunopeptidome from a Genomic Perspective: Establishing the Noncanonical Landscape of MHC Class I–Associated Peptides

**DOI:** 10.1158/2326-6066.CIR-22-0621

**Published:** 2023-03-24

**Authors:** Georges Bedran, Hans-Christof Gasser, Kenneth Weke, Tongjie Wang, Dominika Bedran, Alexander Laird, Christophe Battail, Fabio Massimo Zanzotto, Catia Pesquita, Håkan Axelson, Ajitha Rajan, David J. Harrison, Aleksander Palkowski, Maciej Pawlik, Maciej Parys, J. Robert O'Neill, Paul M. Brennan, Stefan N. Symeonides, David R. Goodlett, Kevin Litchfield, Robin Fahraeus, Ted R. Hupp, Sachin Kote, Javier A. Alfaro

**Affiliations:** 1 International Centre for Cancer Vaccine Science, University of Gdansk, Gdansk, Poland.; 2 School of Informatics, University of Edinburgh, Edinburgh, United Kingdom.; 3 Urology Department, Western General Hospital, NHS Lothian, Edinburgh, United Kingdom.; 4 Institute of Genetics and Cancer, University of Edinburgh, Edinburgh, United Kingdom.; 5 CEA, Grenoble Alpes University, INSERM, IRIG, Biosciences and Bioengineering for Health Laboratory (BGE) - UA13 INSERM-CEA-UGA, Grenoble, France.; 6 Department of Enterprise Engineering, University of Rome “Tor Vergata”, Rome, Italy.; 7 LASIGE, Faculdade de Ciências, Universidade de Lisboa, Lisboa, Portugal.; 8 Division of Translational Cancer Research, Department of Laboratory Medicine, Lund University, Lund, Sweden.; 9 School of Medicine, University of St Andrews, St Andrews, United Kingdom.; 10 Academic Computer Centre CYFRONET, AGH University of Science and Technology, Cracow, Poland.; 11 Royal (Dick) School of Veterinary Studies and The Roslin Institute, University of Edinburgh, Edinburgh, United Kingdom.; 12 Cambridge Oesophagogastric Centre, Cambridge University Hospitals NHS Foundation Trust, Cambridge, United Kingdom.; 13 Translational Neurosurgery, Centre for Clinical Brain Sciences, University of Edinburgh, Edinburgh, United Kingdom.; 14 Department of Biochemistry and Microbiology, University of Victoria, Victoria, Canada.; 15 University of Victoria Genome BC Proteome Centre, Victoria, Canada.; 16 Cancer Research UK Lung Cancer Centre of Excellence, University College London Cancer Institute, London, United Kingdom.; 17 Tumour Immunogenomics and Immunosurveillance Laboratory, University College London Cancer Institute, London, United Kingdom.; 18 Inserm UMRS1131, Institut de Génétique Moléculaire, Université Paris 7, Paris, France.

## Abstract

Tumor antigens can emerge through multiple mechanisms, including translation of noncoding genomic regions. This noncanonical category of tumor antigens has recently gained attention; however, our understanding of how they recur within and between cancer types is still in its infancy. Therefore, we developed a proteogenomic pipeline based on deep learning *de novo* mass spectrometry (MS) to enable the discovery of noncanonical MHC class I–associated peptides (ncMAP) from noncoding regions. Considering that the emergence of tumor antigens can also involve posttranslational modifications (PTM), we included an open search component in our pipeline. Leveraging the wealth of MS-based immunopeptidomics, we analyzed data from 26 MHC class I immunopeptidomic studies across 11 different cancer types. We validated the *de novo* identified ncMAPs, along with the most abundant PTMs, using spectral matching and controlled their FDR to 1%. The noncanonical presentation appeared to be 5 times enriched for the A03 HLA supertype, with a projected population coverage of 55%. The data reveal an atlas of 8,601 ncMAPs with varying levels of cancer selectivity and suggest 17 cancer-selective ncMAPs as attractive therapeutic targets according to a stringent cutoff. In summary, the combination of the open-source pipeline and the atlas of ncMAPs reported herein could facilitate the identification and screening of ncMAPs as targets for T-cell therapies or vaccine development.

## Teaser

Identification of tumor-specific antigens is crucial for developing effective cancer treatments. The authors use *de novo* MS and proteogenomics to generate an atlas of non-canonical MHC class I–associated peptides, providing potential targets for T-cell therapies or vaccines.

## Introduction

The accelerated adoption of mass spectrometry (MS) for high-throughput profiling of immunopeptidomes in cancer has led to several discoveries. Leveraging these studies to improve cancer immunotherapy involves connecting the wealth of immunopeptidomic data to immunogenomics, where the goal is to carefully choose effective targets for T-cell therapies or vaccine development.

The discovery of cancer antigens has mainly focused on mutated tumor-specific antigens (neoantigens) arising from patient-specific somatic mutations. It has been shown that only a small percentage of the numerous nonsynonymous mutations in a tumor actually produce neoantigens (1, 2). The challenging task of identifying those that can evoke a suitable tumor rejection was addressed by Ebrahimi-Nik and colleagues (3). Using a combination of genomics, shotgun MS immunopeptidomics, and targeted MS, they found that (i) MS-identified neoepitopes are a rich source of tumor rejection–mediating antigens, (ii) neoantigens derive from passenger mutations, and (iii) binding affinity and CD8^+^ T-cell responses in tumor-bearing hosts are poor predictors of antitumor activity *in vivo*. Although neoantigens confer an advantage to patients undergoing immunotherapy (4), their patient-specific nature is a major bottleneck when producing off-the-shelf treatments for a large number of individuals. Alternatively, shared neoantigens (ref. 5; i.e., recurrent mutations in cancer) could offer a new line of population-level immunotherapy. However, high-throughput experimental profiling of such broadly presented neoantigens across the human population is a long-term goal with many milestones to be achieved.

Recently, tumor antigens that exceed the exome boundaries (i.e., noncanonical) have attracted attention as potential targets as a result of their immunogenicity and recurrence among cancer patients (6). These antigens find their way to the cell surface through rapid degradation (7) of “noncoding” translation products stemming from novel open reading frames (nORF; ref. 8). In addition, “noncoding” translation products can originate from other sources (9), including intron retention (IR; ref. 10), ribosomal slippage (11), and frameshift mutations (12). In 2016, Laumont and colleagues (13) demonstrated their association with MHC molecules using a reductionist approach based on 6-frame translation and subsequently their recurrence between patients (14). Ribo-Seq has proven to be an immensely valuable tool for identifying noncanonical MHC class I–associated peptides (ncMAP) as it provides experimental evidence for their noncanonical translation and MHC class I presentation when combined with MS immunopeptidomics (6, 15, 16). Despite previous efforts to study noncanonical immunopeptidomes, the requirements of such multi-level experimental data (Ribo-seq and/or RNA sequencing) or computational struggles when dealing with large MS databases have hindered their large-scale profiling in a harmonized manner across multiple cancer types from hundreds of samples.

With these considerations in mind, we developed Closed Open *De novo* – deep immunopeptidomics pipeline (COD-dipp), a pipeline based on deep learning *de novo* MS to enable the discovery of ncMAPs. Owing to the potential involvement of posttranslational modifications (PTM) in this process (1), we added an open search component for their discovery. We applied COD-dipp to a large-scale dataset using immunopeptidome profiles of over 772 samples from 26 (1, 2, 13, 14, 17–39) published studies and 11 cancer types. We identified a range of PTMs of potential interest from a therapeutic standpoint and tackled the noncanonical immunopeptidome. We validated the *de novo* identified ncMAPs and controlled their FDR to 1% using a second-round search with tuned PTM parameters, in addition to a series of quality-control steps. Our large-scale analysis revealed 8,601 ncMAPs, accounting for 1.7% of immunopeptidomes. These peptides had varying levels of tumor selectivity, defined by their parent gene expression levels in normal tissues. We suggest 17 ncMAPs as attractive therapeutic targets using a stringent tumor-selectivity cutoff.

## Materials and Methods

### Dataset selection

Twenty-four studies were selected on the basis of a list of keywords related to immunopeptidomics (Supplementary Method S1). Low-resolution analyses were eliminated, and MHC class I–related datasets conducted with at least one of the following instruments were kept: Q Exactive, Q Exactive plus/HF/HFX, LTQ Orbitrap Velos, LTQ Orbitrap Elite, Orbitrap Fusion, and Orbitrap Fusion Lumos (Supplementary Table S1). An additional study was considered from the MassIVE (RRID:SCR_013665) database, as it incorporates 95 HLA-A, -B, -C, and -G mono-allelic cell lines (28, 39). An auxiliary immunopeptidomic dataset (38) covering 30 healthy tissues from 21 healthy individuals was also used to partly assess cancer selectivity.

### Proteogenomic database generation

#### Canonical protein database for MS database search

A protein database was downloaded using ENSEMBL r94 BioMart (RRID:SCR_002344); decoy sequences were appended by reversing the target sequences, and 116 contaminant proteins were added (40).

#### Noncanonical protein database for alignment using BLAST-like alignment tool

A pre-mRNA 3-frame translation (3FT) database was generated from genes with a protein-coding biotype based on ENSEMBL r94 (RRID:SCR_002344) using the AnnotationHub and Biostrings (RRID:SCR_016949) R packages.

#### COSMIC mutated protein database for BLAST-like alignment tool alignment

COSMIC (RRID:SCR_002260) coding Mutants (41) VCF v95 was downloaded along with ENSEMBL v94 CDS and GTF files. An in-house Python (RRID:SCR_008394) package was used along with the previously mentioned inputs to generate a FASTA file containing the corresponding mutated protein sequences.

### MS computational analysis

Algorithms representing three main philosophies of peptide-spectrum matching including open search, *de novo* sequencing, and closed search were used. The open search approach allowed the identification of distantly related peptides and could identify PTMs and single amino acid variations. The *de novo* sequencing approach derived sequences from first-principle analysis of the MS^2^ spectra. The closed search approach, used as a validation step, assumed a specific set of reference protein sequences and allowed for limited PTMs. Although each approach has its own limitations, our strategy addressed them by combining a closed search with a *de novo* sequencing approach and implementing multiple filtering steps for accuracy control and quality control checkpoints (see Supplementary Fig. S1).

#### Data conversion

The proprietary RAW files acquired from the selected instruments were converted to mzML and MGF formats using msconvert (ProteoWizard version 3.0.19295. c8b8b470d, RRID:SCR_012056) with the peak-picking and TPP compatibility filters.

#### Open search analysis

The MSFragger (42) v2.2 search engine was used to conduct an open search analysis against the ENSEMBL r94 protein database in combination with PTMiner (43) v1.1.2, to apply a transfer FDR and a false localization rate of 1% (FLR, the rate of falsely localizing the site of modification). Unspecific cleavage generating peptides 8 to 25 amino acids long with no fixed/variable PTMs was considered. Further analysis revealed that the frequent unexplained mass shifts observed during the open-search annotations were caused by nonspecific cleavage. To address this issue, an open-search postprocessing algorithm, PTMiner, was employed to effectively corrects for mass shifts introduced by in-source fragmentation, nonspecific digestion, or missed cleavage, by adding or deleting amino acids from the peptide N- or C-termini. For instance, a deviation of −128.1 to −128.08 Dalton on lysine residues was frequently detected on the first 2 or last 2 amino acids of peptides. The deviation was caused by nonspecific cleavage during the open search and resulted in an incorrect assignment of a negative mass shift of a lysine due to the presence of an additional lysine in the sequence. As these cases are not biologically meaningful, unexplained mass shifts were removed from the final results of the study.

#### *De novo* analysis

DeepNovoV2 (44) is a neural-network-based *de novo* peptide sequencing model that integrates convolutional neural networks and long short-term memory (LSTM) architectures. This deep-learning design extracts features from both the spectrum and the language of the presented peptides. DeepNovo has demonstrated improved performance compared with the state-of-the-art *de novo* sequencing algorithms by large margins (44). The model can be tuned on a restricted peptide space to improve its performance. The training, testing, and validation sets were derived from MS-GF+ (v2019.04.18, RRID:SCR_015646) database search results for each sample. The search used the ENSEMBL v94 protein database and 8 to 25 amino acid peptides with unspecific cleavage, no fixed/variable PTMs and an FDR of 1% applied by Scavager (45). The trained models were used to perform *de novo* (prediction) on the remaining unmatched spectra of each sample (from MS-GF+ after 1% FDR control). Accuracy was calculated by comparing the *de novo* predicted sequences and MS-GF+ results on the validation set. *A de novo* score threshold that controlled the accuracy at 90% within the validation set was applied to the predicted sequence in a sample-specific manner.

#### *De novo* peptide annotation

*De novo* peptides from canonical human proteins were identified using BLAST-like alignment tool (BLAT; ref. 46; RRID:SCR_011919) alignment against the target-decoy protein database. Sequences perfectly matching any protein sequence were considered exonic (one mismatch allowed for the isobaric amino acids leucine and isoleucine). All remaining sequences unexplained by proteins were considered potential noncanonical peptides and were aligned against the pre-mRNA 3FT database. Stringently, peptides perfectly matching a 3FT sequence without any mismatch were required to have at least three mismatches with any known protein sequence before being considered noncanonical. Because peptide-spectrum matches (PSM) can be assigned without complete sequencing accuracy, requiring a 3 amino acid difference alongside the 90% accuracy cutoff above increases the confidence that the peptides assigned fall far outside the standard human proteome. Remaining *de novo* peptides without any canonical or noncanonical annotation were labeled as ‘unmapped peptides’ and discarded.

#### Second-round search

A second-round search was performed using the FragPipe (40, 42) headless pipeline, which includes MSFragger v3.4, MSBooster (bioRxiv 2022.10.19.512904), and Philosopher (40). Noncanonical peptides from all samples were concatenated with the ENSEMBL v94 protein into a custom database. Only four of the most abundant PTMs were considered to avoid a large search space complexity, inflated FDR, and decreased sensitivity. The following variable PTMs were included: methionine oxidation, N-terminal acetylation, cysteinylation, and cysteine carbamidomethylation (for samples treated with iodoacetamide). Unspecific cleavage generating peptides 7 to 15 amino acids long was considered. The ion, PSM, and peptide-level FDR were maintained at 1%.

### Alignment of immunopeptides to the genome

Second-round search noncanonical peptide coordinates were retrieved from the 3FT database FASTA headers and stored in BED format.

### Open reading frame analysis

Upstream genomic sequences of ncMAPs were scanned for start codons up to the first encounter with a stop codon. Sequences were centered around the detected start codons and stretches of 100 nucleotides from each side were extracted. Translation initiation site (TIS) scores were predicted for each sequence using TITER (47), a deep-learning-based framework for accurately predicting TIS on a genome-wide scale based on QTI-seq data. A TIS score greater than 0.5 was considered a positive prediction.

### IR analysis

For each intron in the UCSC hg38 KnownGene table (RRID:SCR_005780), the first codon coordinates of the corresponding upstream exon in-frame with the canonical translation were extracted and stored in BED format (see **Pseudocode 1**). Intronic coordinates from the generated BED file were intersected with the ncMAPs BED file using pybedtools (ref. 48; RRID:SCR_021018). Intronic retention events were considered possible when ncMAPs within introns were in-frame with their upstream exons (see **Pseudocode 2**).

**// Pseudo-code 1: extracts the start coordinate of the first in-frame codon for each exon (inframeCoordinate variable)**

for each transcript

 remainderValue = θ

 for each exon

  if strand is positive

   if downstream intron exists

leftoverBases = remainder of (ExonEndCoordinate - remainderValue - ExonStart + 1) / 3

    if remainderValue is equal to θ

     inframeCoordinate = ExonStartCoordinate

else

 inframeCoordinate = ExonStartCoordinate – remainderValue

if leftoverBases is greater than θ

 remainderValue = 3 - leftoverBases

addToTable(transcript, chromosome, ExonStart, ExonEnd, inframeCoordinate, IntronStart, IntronEnd)

if strand is negative

 if downstream intron exists

  leftoverBases = remainder of (ExonSart - ExonEndCoordinate + remainderValue + 1) / 3

 if remainderValue is equal to θ

  inframeCoordinate = ExonEndCoordinate

 else

  inframeCoordinate = ExonEndCoordinate + remainderValue

 if leftoverBases is greater than θ

  remainderValue = 3 - leftoverBases

addToTable(transcript, chromosome, ExonStart, ExonEnd, inframeCoordinate, IntronStart, IntronEnd)

**// Pseudo-code 2: checks if each intronic ncMAP is in-frame with its upstream exon.**

ncMAPIsInFrame = False

if strand is positive

 // firstCoordinate = start coordinate of ncMAP

 // secondCoordinate = start coordinate of the first inframe codon from previous exon

 coordinateDifference = firstCoordinate – secondCoordinate

 if remainder of (coordinateDifference / 3) is equal to θ

  ncMAPIsInFrame = True

else:

 // firstCoordinate = start coordinate of first inframe codon from previous exon

 // secondCoordinate = end coordinate of ncMAP

 coordinateDifference = firstCoordinate – secondCoordinate

 if remainder of (coordinateDifference / 3) is equal to θ

  ncMAPIsInFrame = True

### Frameshift mutation analysis

The COSMIC (41) v95 coding mutations (RRID:SCR_002260) VCF file was downloaded and converted into a protein FASTA file using a VCF-to-Proteogenomics toolkit (https://github.com/immuno-informatics/VCFtoProteogenomics) ncMAPs were then aligned to the resulting 16 GB FASTA using BLAT v35 (46). Only hits with exact matches to sequences from frameshift mutations were considered.

### Comparison of the identified ncMAP between studies

ncMAPs from 4 different studies (6, 13, 16, 49) were collected. First, sequences were aligned to the human proteome (ENSEMBL v94) using BLAT v35 (46). Sequences found in human proteins were discarded, and the remaining sequences were aligned to the 3FT database with one mismatch allowance for the isobaric amino acids leucine and isoleucine, as allowed for COD-dipp ncMAPs. Genomic coordinates of the sequences found in the 3FT database were extracted and overlapped between studies using the ChIPpeakAnno (50) R package (RRID:SCR_012828). A minimum overlap of 21 nucleotides (7 amino acids) between two sequences was required.

### Cancer selectivity of the ncMAP

Tumor specificity has been previously implied when peptide parent genes are either completely absent or present in trace amounts in healthy tissues (6, 14, 16) because MHC class I presentation is preferentially derived from highly abundant transcripts (28, 30). While tumor specificity implies the expression of an antigen solely in tumor samples, the experimental design of this study cannot guarantee this constraint. Instead, cancer-selective ncMAPs were conservatively identified through three iterative steps:

#### Step 1: panel of normal immunopeptidomes

In addition to the 88 healthy MS samples from the initial set of the 25 considered studies, the HLA Ligand Atlas (38) was used to extend the panel of normal immunopeptidomes and partly assess the cancer selectivity of the 8,601 identified ncMAPs. The HLA Ligand Atlas is a pan-tissue immunopeptidomic reference for 30 healthy tissue types obtained from 21 human subjects. The resulting 334 healthy samples (see Supplementary Table S1) were analyzed in the same manner as in the second-round search (see *Second-round search* above). ncMAPs identified in the panel of normal immunopeptidomes were labeled as non-cancer selective.

##### Dimensionality reduction of the HLA-binding motif space:

Binding affinity prediction was employed to identify similarities and differences in HLA-binding motifs among the 65 healthy and 51 tumor-only HLA alleles. NetMHCpan-4.1 was used to evaluate the binding of 1,000,000 random peptides to each allele, which resulted in a binding matrix (BM) of 116 alleles and 1,000,000 peptides. A value of 1 was assigned to strong binders (EL rank ≤ 0.5%) in the BM; otherwise, a value of 0 was assigned. A pairwise cosine distance matrix (DM) was then calculated to assess the similarity of binding between alleles. The DM was then reduced using t-SNE to visualize the data in 2D with a perplexity of 20 and 500 iterations.

#### Step 2: parental gene expression levels in healthy tissue

The gene expression levels of the identified ncMAPs were retrieved from the GTEx v8 (51) dataset, consisting of 29 tissues from 948 healthy donors and 17,382 overall samples. Considering all individuals, the 90th percentile value of normalized expression was assigned to each gene per tissue as a strict step to guarantee the upper-end gene expression in healthy tissues. A stringent cutoff for cancer selectivity was used to shortlist ncMAPs whose parent genes fell below a 1 TPM expression cutoff (excluding the testis tissue given its immune-privileged status). It is worth noting that this stringent threshold removes 92% of protein-coding genes that show expression above 1 TPM in any tissue within the GTEx v8.

#### Step 3: protein expression levels in healthy tissue

The protein expression levels of ncMAPs passing the 1 TPM cutoff were retrieved from the Human Protein Atlas V22.0 database (52). ncMAPs without parent protein expression in healthy tissues were labeled as cancer-selective (excluding the testis tissue given its immune-privileged status).

### Code availability

The COD-dipp code, intended for high-performance computing, is available on the GitHub repository: https://github.com/immuno-informatics/COD-dipp.

### Data availability

The data analyzed in this study were obtained from PRIDE at PXD004746, PXD014017, PXD012308, PXD011628, PXD012083, PXD011766, PXD013057, PXD011723, PXD007203, PXD004233, PXD003790, PXD001898, PXD007860, PXD011257, PXD007935, PXD009749, PXD009753, PXD009750, PXD009751, PXD009752, PXD009754, PXD009755, PXD004023, PXD007596, PXD009531, PXD010808, PXD008937, PXD009738, PXD006939, PXD005231, PXD000394, PXD004894, PXD019643 and from massIVE at MSV000080527, MSV000084172, MSV000084442. The results of this study are available within the article and its supplementary data files and are accessible on the following figshare repository: https://doi.org/10.6084/m9.figshare.16538097.

## Results

### Immunopeptidomic MS datasets

**Figure 1.**
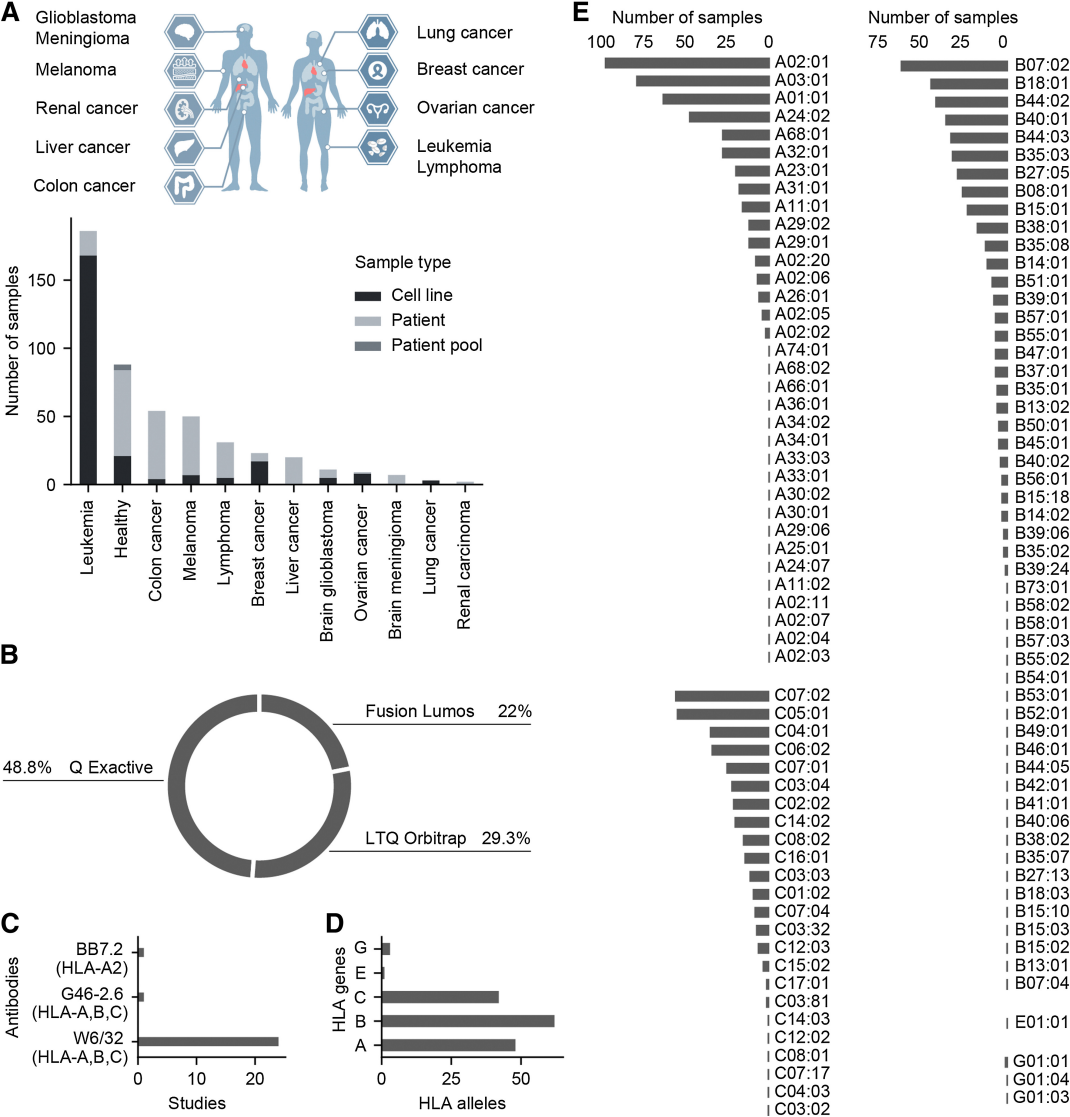
Infographics of immunopeptidomic datasets included in this study. **A,** Different types of cancer considered in this study with the number of samples and sample types per cancer type. **B,** Proportions of different MS instruments used in this study. **C,** Antibodies used for IP. **D,** Overall count of HLA alleles per HLA gene. **E,** Overall count of MS immunopeptidomic samples per HLA allele.

We selected 25 immunopeptidomic MS studies (see Supplementary Table S1) to create a cancer-centered dataset of MHC class I presentation. Data-dependent acquisition (DDA) studies covered eleven cancer types distributed across the brain (Glioblastoma and Meningioma), lung, skin, liver, blood (Leukemia and Lymphoma), colon, ovaries, kidneys, and breast. Moreover, tumor and healthy samples were derived from either cell lines or patient tissues (Fig. 1A and Supplementary Method S1). We selected publicly available studies with data generated using high-resolution MS instruments (LTQ Orbitrap, Q Exactive Plus/HF/HFX, and Fusion Lumos) to minimize the bias associated with older tandem MS instrumentation (Fig. 1B). Within our dataset, the most commonly used monoclonal antibody for HLA class I immunoprecipitation (IP) was W6/32 in comparison with the other antibodies (BB7.2 and G46–2.6; Fig. 1C, see Supplementary Table S1). The selected studies covered five different HLA class I genes, with HLA-A, B, and C being the most studied compared with HLA-E and -G (Fig. 1D). Furthermore, the included MS samples covered 114 HLA alleles (Fig. 1E).

### COD-dipp

**Figure 2.**
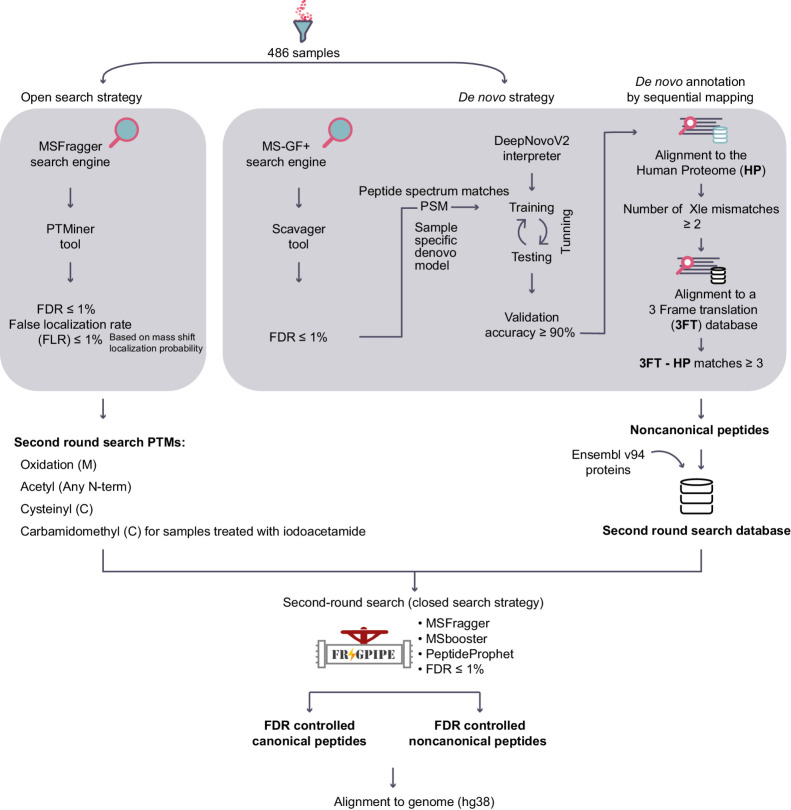
COD-dipp: A new high-throughput pipeline for a deep interrogation of immunopeptidomic datasets. Samples are first analyzed with an open search strategy to detect the landscape of PTMs. An FLR for the PTMs and FDR of 1% are applied. Simultaneously, the samples are analyzed using a novel *de novo* approach to identify noncanonical peptides. The *de novo* strategy trains a model per sample using quality-controlled PSMs from the MS-GF+ search engine to learn the direct interpretation of sample-specific mass spectra. The MS-GF+ results are split into three groups: training and testing to tune the hyperparameters and account for overfitting, and a validation group to approximate the accuracy per sample. *De novo* predicted peptides with an accuracy of at least 90% are sequentially mapped against the Human proteome (HP) then a 3-frame translation (3FT) database of protein-coding genes (1 mismatch allowed between leucine/isoleucine, i.e., Xle). Predicted *de novo* peptides matching any known protein are labeled “canonical”. Peptides mapping to the 3FT database with at least 3 amino acids mismatches from any known protein sequence are labeled “noncanonical”. Finally, a second-round search is performed as a validation approach. Four of the most abundantly identified PTMs and a custom database consisting of ENSEMBL proteins and noncanonical peptides are considered. The resulting canonical and noncanonical peptides are controlled to an FDR of 1% and aligned to the hg38 human genome.

We present COD-dipp, an open-source high-throughput pipeline with novel postprocessing steps, to deeply interrogate immunopeptidomic datasets (Fig. 2). We used this pipeline to screen for ncMAPs in datasets using DDA due to its widespread use. To identify posttranslationally modified MHC class I–associated peptides (ptmMAP), we performed an open-search analysis with MSFragger (42) and controlled both FDR and the FLR to 1% with PTMiner (43). To identify ncMAPs, we used DeepNovoV2 (44) for *de novo* analysis. In combination with the PSM level information of MS-GF+ (53), DeepNovoV2 was trained to interpret the raw MS data in a sample-specific manner. The training step for such a deep learning approach is crucial for learning the features of tandem mass spectra, fragment ions, and leveraging sequence patterns in the immunopeptidome to impute missing MS^2^ fragments. All high-quality *de novo* peptides (90% accuracy) were sequentially mapped (46) to the human reference proteome to reveal the *de novo*-based canonical MHC class I–associated peptides, and to a 3FT database to reveal the *de novo*-based ncMAPs. Finally, an orthogonal validation step was performed by a second-round search to control a 1% FDR for the *de novo* identified ncMAPs while considering the most abundant PTMs found by the open-search strategy. Applying the COD-dipp pipeline across the dataset revealed the breadth of ptmMAPs and ncMAPs.

### ptmMAPs

**Figure 3.**
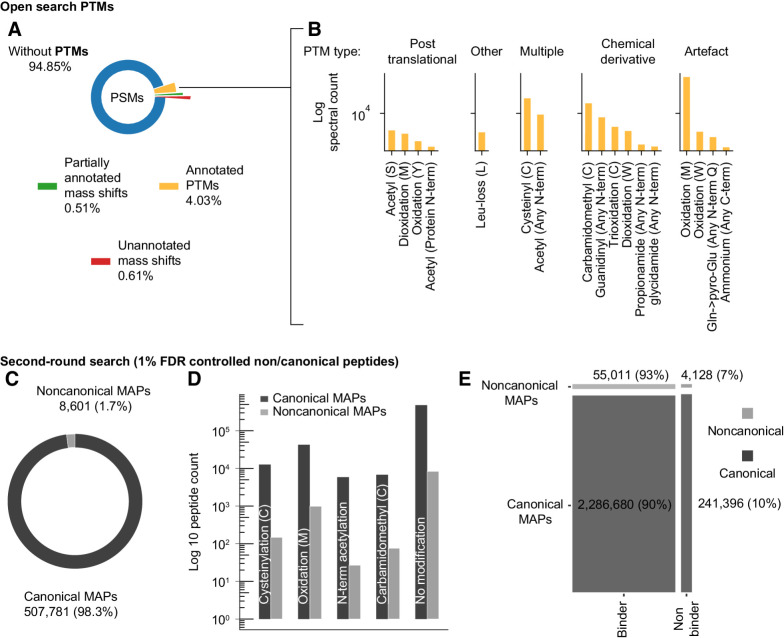
Landscape of posttranslationally modified and ncMAPs. Open search: **A,** Overview of PTMs identified by open search (blue: spectra without PTMs, orange: spectra with a known UNIMOD PTM localized on a specific amino acid on the peptide. Green: The mass shift is localized, however the known PTM options do not fit the modified residue. Red: Otherwise). **B,** Most abundant “annotated PTMs” grouped by type. Second-round search: **C**, Fraction of canonical (dark gray) and noncanonical (light gray) MAPs in the immunopeptidome. **D,** Proportion of canonical (dark gray) and noncanonical (light gray) MAPs with/without PTMs. **E,** Fraction of binders versus nonbinders for both canonical and noncanonical MAPs using NetMHCpan 4.1.

The open search analysis reported 4.03% of the MS spectra showing PTMs (Fig. 3A). Some identified PTMs were confirmatory, representing chemical modifications from sample preparation methods (cysteine carbamidomethylation) or common chemical derivatives (methionine oxidation and di-oxidation). We also observed PTMs that are extremely common in proteins, such as protein N-terminal acetylation, affecting multiple properties such as half-life time, folding, and interaction. On the other hand, some of the identified PTMs have been reported previously to increase immunogenicity against diseases (54) and protect against degradation [tri-oxidation of cysteine (55), cysteinylation (56), and N-term serine acetylation, see Fig. 3B and Supplementary Table S2]. Furthermore, 1.12% of spectra from open search showed unknown mass shifts, as illustrated in Fig. 3A (green and red). This category was partly populated by computational artifacts and was excluded from the final results. To validate these findings, we performed an independent post-search by crosschecking the identifications from our open search with those of the original studies. The results showed 96.1% agreement in PSMs, which are detailed in Supplementary Method S2: Validation 1 and Supplementary Fig. S2.

### ncMAPs

We explored the ncMAP landscape in cancer using our workflow (Fig. 2) and identified 10,413 unique *de novo*–based ncMAPs from intragenic noncoding regions (before the second-round search validation), which accounted for 3.7% of the identified *de novo* sequences. We took two additional validation steps, including checking the identification scores as well as the correlation between the experimental and theoretical liquid chromatography retention times, to guarantee the correctness of these identifications (see Supplementary Method S2: Validation 2 and 3, and Supplementary Fig. S2). The *de novo* noncanonical peptides showed strong evidence of high-quality identification (i.e., correctly predicted complete peptide sequences). Even with this strong evidence, it was possible that chromatic behavior remained unchanged in certain instances where neighboring amino acids were in flipped positions, or that a 90% accuracy rate still led to an uncertain FDR percentage. Hence, we confirmed the identified 10,413 *de novo*–based ncMAPs by performing a second-round search for additional validation and controlling the FDR at 1%. Several PTMs were also considered in the parameters from the *a priori* knowledge provided by the open search strategy. Of the 516,382 uniquely identified peptides in the second-round search, 1.7% (8, 601) were noncanonical (Fig. 3C and Supplementary Table S3). The PTM profiles (Fig. 3D) of canonical (dark gray) and noncanonical (light gray) peptides appeared to be similar, with M oxidation being the most prevalent modification. The identified ncMAPs showed comparable spectra from patients within the same studies and from different studies (Supplementary Figs. S3, S4, and S5 provide examples of such similarities). The binding affinities of all 8,601 ncMAPs resulting from the second-round search were further investigated using NetMHCpan 4.1 (57). The binding prediction analysis showed a comparable binding rate for both the canonical (90%) and noncanonical (93%) MAPs, as depicted in Fig. 3E. We further took four additional independent post-search validation steps, including checking retention time shifts induced by PTMs, mass accuracy, and spectra comparison with those of the original studies, guaranteeing the correctness of the ncMAPs identified by the second-round search (see Supplementary Method S2: Validation 4, 5, 6, and 7, and Supplementary Fig. S2).

#### Comparison of COD-dipp ncMAPs with the literature

**Figure 4.**
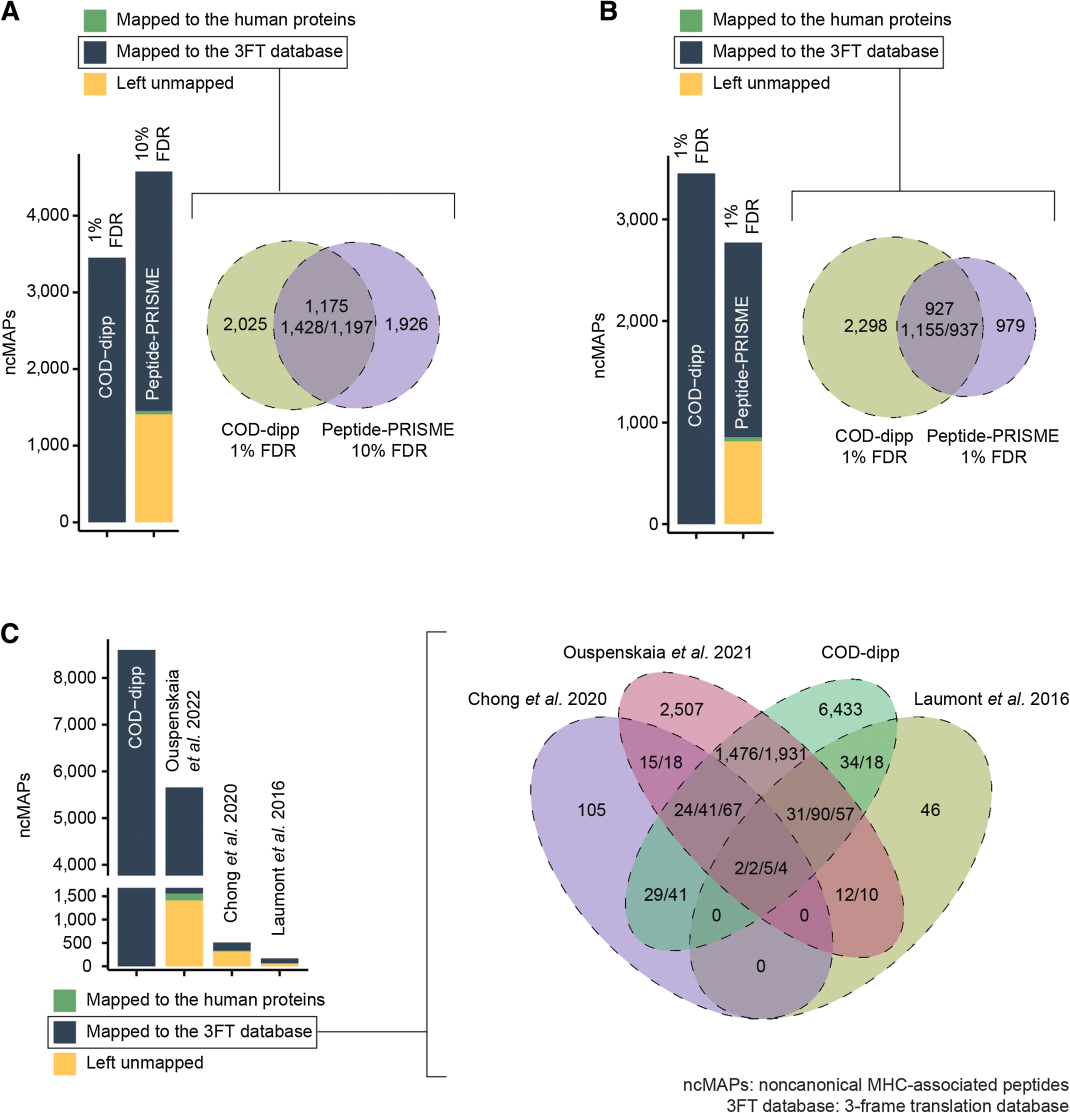
Comparison of COD-dipp ncMAPs with other studies. Because the COD-dipp ncMAPs are restricted to the 3-frame translation (3FT) of protein-coding genes, sequences from the literature were aligned to the same 3FT database for comparison purposes. The intersection is based on genomic coordinates to deal with sequences that partially match (i.e., longer, shorter, or partially overlapping). Because the Venn is generated by overlapping genomic coordinates of the ncMAPs, the original counts for each study are listed from left to right (i.e., on the right-hand side of panel C, the notation 29/41 refers to 29 instances for Chong and colleagues 2020 and 41 for COD-dipp). **A,** Comparison with peptide-PRISM published ncMAPs at a 10% FDR. COD-dipp ncMAPs were restricted to 3 studies in common with Erhard and colleagues 2020. **B,** Comparison with peptide-PRISM published ncMAPs at a 1% FDR. COD-dipp ncMAPs were restricted to 3 studies in common with Erhard and colleagues 2020. **C,** Comparison of the atlas of ncMAPs revealed by COD-dipp to 3 previous studies.

To assess the performance of our COD-dipp method, we conducted a comparison with the results of peptide-PRISME by Erhard and colleagues 2020 (49). Our comparison was based on three common studies (1, 14, 34) and resulted in 3,453 at 1% FDR from COD-dipp along with 4,576 ncMAPs at 10% FDR from Erhard and colleagues. We first aligned Erhard and colleagues*’* ncMAPs to the human proteome and eliminated a small fraction (1.4%) that matched the canonical protein sequences (Fig. 4A, left-hand side). Because the COD-dipp ncMAPs were restricted to the 3FT of protein-coding genes, we aligned the remaining ncMAPs from Erhard and colleagues to the same 3FT database for comparison purposes. Fig. 4A (left-hand side) shows that 68.25% of ncMAPs were successfully mapped to the 3FT database. The rest (30.35%) that did not align to any of the human proteome or the 3FT database are shown in yellow on Fig. 4A left-hand side. This unmapped fraction consisted of ncMAPs from regions of the genome not studied herein, such as intergenic regions, antisense translation, etc. The successfully mapped fraction to the 3FT database (navy) of 3,123 ncMAPs along with 3,453 ncMAPs from COD-dipp were then compared, as shown in Fig. 4A right-hand side (see Supplementary Table S4). peptide-PRISME shared 38% (1,197) of its ncMAPs (intersection) with COD-dipp (Fig. 4A right-hand side) and showed 62% (1,926) of exclusive ones. Adjusting the higher FDR used by peptide-PRISME from 10% to 1% increased the shared fraction to 48.9% (Fig. 4B), along with a ∼2.4-fold decrease in total ncMAPs (from 4,576 to 1,916). At an FDR of 1%, COD-dipp identified 2.34 times more exclusive ncMAPs (2,298 vs. 979) from the 3FT of protein-coding genes.

To contextualize our findings from COD-dipp within the existing literature on ncMAPs, we compared our results with those of three previous studies: (i) Laumont and colleagues 2016 (13), (ii) Chong and colleagues 2020 (6), and (iii) Ouspenskaia and colleagues 2021 (16), as shown in Fig. 4C. We used the same mapping procedure that was applied to peptide-PRISME results. We eliminated a fraction of sequences mapping to known proteins, which was 4%, 5%, and 3% of sequences for Chong and colleagues 2020, Laumont and colleagues 2016, and Ouspenskaia and colleagues 2021, respectively (see Fig. 4C left-hand side). Fig. 4C left-hand side shows in navy the fractions of ncMAPS that were successfully mapped to the 3FT database, which was 34.38% for Chong and colleagues 2020, 63.69% for Laumont and colleagues 2016, and 72.74% for Ouspenskaia and colleagues 2021. The remaining ncMAPs that did not align (Fig. 4C left-hand side in yellow) to any of the human proteome or the 3FT database originate from sources not studied herein. For instance, Laumont and colleagues 2016 included 6-frame translation in their MS search database, which accounts for intergenic regions, antisense translation, long noncoding RNA, and retroelement sources. Both Chong and colleagues 2020 and Ouspenskaia and colleagues 2021 added Ribo-Seq detected proteins to their MS database searches, accounting for all possible nORFs, even those outside of known genes. The fractions successfully mapped to the 3FT database (navy) from these three studies, along with the 8,601 ncMAPs from COD-dipp, were then compared, as shown in Fig. 4C right-hand side (Supplementary Table S4). Intersections with COD-dipp were 31.42% for Chong and colleagues 2020, 38.3% for Ouspenskaia and colleagues 2021, and 45.8% for Laumont and colleagues 2016, respectively. In contrast, intersections with all other studies were 40% for Chong and colleagues 2020, 38.66% for Ouspenskaia and colleagues 2021, and 65.93% for Laumont and colleagues 2016. Hence, COD-dipp ncMAPs alone accounted for 78.55% of Chong and colleagues 2020’s intersection, 96.07% of Ouspenskaia and colleagues 2021’s intersection, and 69.47% of Laumont and colleagues 2016’s intersection. COD-dipp ncMAPs accounted, on average, for 81.36% of the intersection when comparing three previously published ncMAP sets, thus validating our approach. With 2,168 ncMAPs (25%) shared with the literature and 6,433 new ncMAPs, we have revealed an atlas of noncanonical MHC class I presentation.

#### Properties and origins of ncMAPs

**Figure 5.**
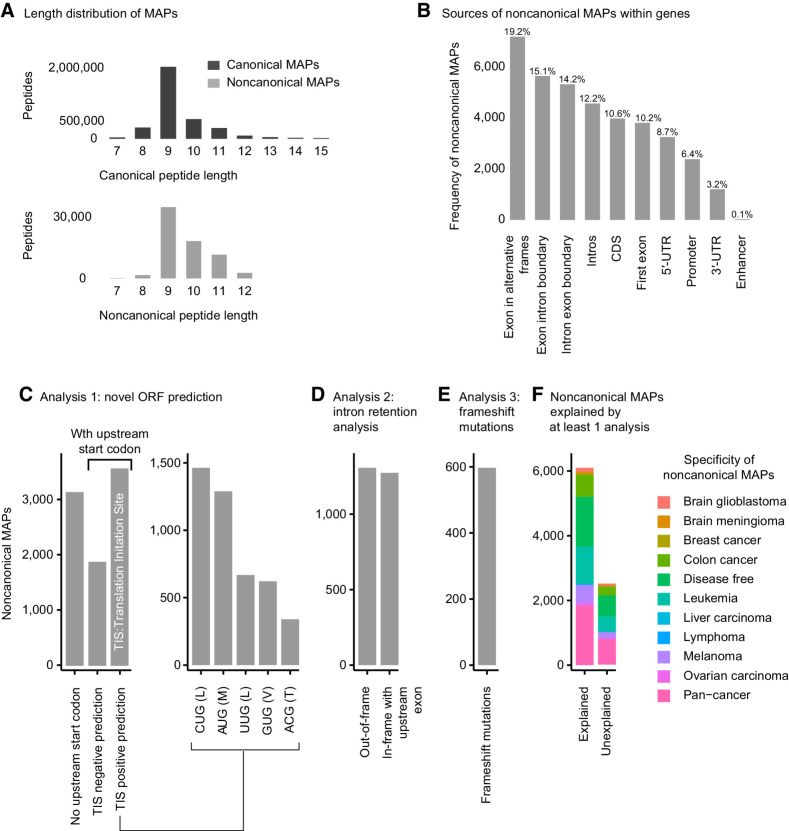
Origins of ncMAPs. **A,** Peptide length distribution of canonical (dark gray) and noncanonical (light gray) MAPs. **B,** Annotation of ncMAPs across gene features. **C,** Analysis of ncMAPs that could originate from nORF. Upstream start codons of noncanonical MAPs are analyzed for their potential to initiate translation and produce ORFs (left-hand side) as a source of ncMAPs. The frequencies of different start codons for positively predicted TIS are shown on the right-hand side. **D,** Analysis of ncMAPs from intronic regions that may originate from IR events. Translation of MAPs from IR sources should be in-frame with the corresponding upstream exons. **E,** Analysis of ncMAPs that could originate from frameshift mutations in cancer. ncMAPs are aligned to an in-silico translated protein database of COSMIC somatic frameshift mutations. **F,** Summary indicating whether the ncMAPs can be accounted for by any of the analyses conducted in panels **C**, **D**, or **E**.

We compared the sequence lengths of canonical and noncanonical MAPs (Fig. 5A) and found them to be similar, with a slight skew of the noncanonical category toward longer lengths. This could be due either to an actual preference of ncMAPs toward longer sequence lengths or simply the consequence of requiring 3 amino acid differences from any known proteins favoring longer sequences. Next, we inspected ncMAPs according to their relative positions within protein-coding genes (Fig. 5B). Exonic regions translated in alternative frames were the main source of ncMAPs (19.2%). These events could arise from frameshift mutations, initiation codon readthrough (58), nORFs, or ribosomal slippage (11) during translation (i.e., ribosome frameshifting). Intronic regions were the second most abundant source of ncMAPs (12.2%). These events can arise from frameshift mutations, nORFs, or IR. Interestingly, 5′-UTRs contributed to 10.2% of ncMAPs and have been shown to produce translation products through upstream ORFs along with a non-AUG start codon (59). Finally, 3′-UTRs contributed the least toward ncMAPs (3.2%), potentially through stop codon read-through (60). It is important to note that these categorizations are not mutually exclusive and that an ncMAP sequence may have multiple assignments due to the overlapping nature of transcripts. We conducted three analyses to estimate how well the nORFs (i), IR (ii), and frameshift mutations (iii) could explain the detected ncMAPs. (i) ncMAPs with upstream start codons (AUG, CUG, UUG, GUG, and ACG) accounted for 63.4%, and 41.5% were predicted to be TIS using TITER (Fig. 5C, left-hand side; ref. 47). The breakdown of the TIS start codon distribution (Fig. 5C, right-hand side) showed CUG (L) as the most abundant nORF start codon, and 70% of the predicted TIS showed non-AUG start codons, in line with previous findings (15). (ii) Translation frames of ncMAPs from intronic regions were checked for compatibility with upstream exons, and 49.4% were found in-frame with upstream exons, making IR events a possible source (Fig. 5D). (iii) A total of 597 ncMAPs were found in aberrant proteins from frameshift mutations in cancer (Fig. 5E and Supplementary Table S5; ref. 41). Eventually, 70.1% of ncMAPs were explicable by novel ORFs, IR, or frameshift mutations (Fig. 5F). ncMAPs were found to be presented by all 113 alleles in our dataset, except for the HLA-C*07:17 allele, mostly because of low sample coverage by MS for this allele (see Supplementary Fig. S6). Furthermore, the average noncanonical presentation per HLA supertype (61) was 1%, except for A03, which was 5% (see Supplementary Fig. S6).

#### Cancer selectivity of ncMAPs

**Figure 6.**
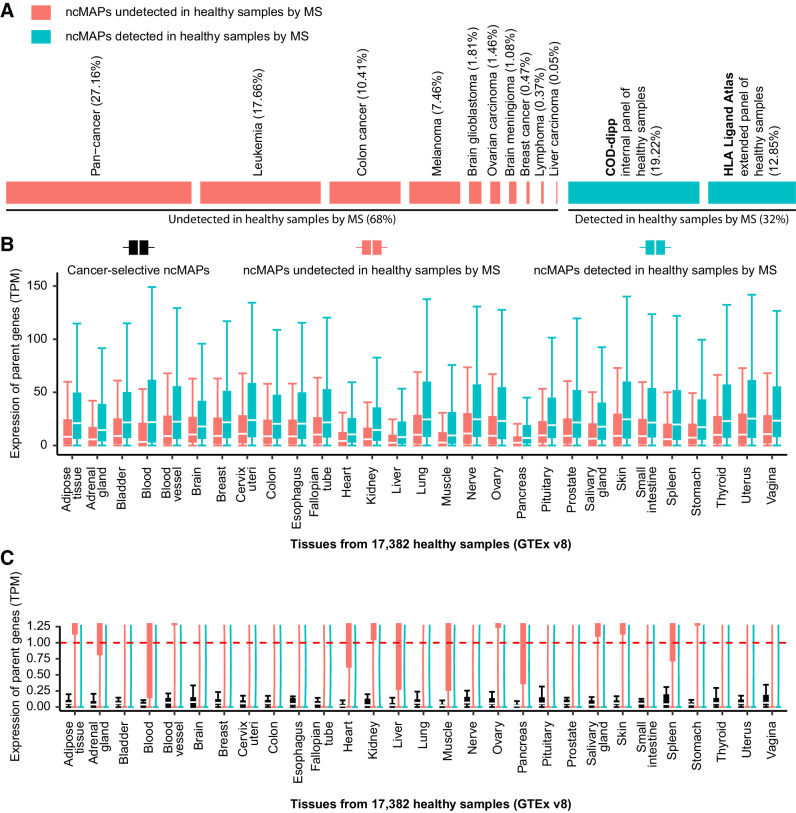
Cancer selectivity of ncMAPs. **A,** Percentage of ncMAPs that were solely in healthy and/or tumor samples by MS (blue) and ncMAPs undetected in healthy samples by MS (red). **B,** Parent gene expression of ncMAPs in TPM in 29 healthy tissues from 17,382 samples (GTEx v8 dataset). ncMAPs are distributed over two groups: ncMAPs detected in healthy samples by MS in blue, and ncMAPs undetected in healthy samples by MS in red. **C,** Parent gene expression of ncMAPs in TPM in 29 healthy tissues from 17,382 samples (GTEx v8 dataset). A limit on the gene expression (y-axis) of 1.2 TPM was set to visualize cancer-selective ncMAPs in black.

Of the 8,601 identified ncMAPs, 2,758 were detected in the panel of normal healthy tissue by MS and were labeled as noncancer–selective. The panel of normals originally consisted of healthy MS samples from all considered studies, which we extended by adding the HLA Ligand Atlas (38), a pan-tissue immunopeptidomic reference of 30 healthy tissue types obtained from 21 human subjects. Fig. 6A shows the ability of the extended panel of normals to capture several more ncMAPs (12.85%) in healthy tissues that were not observed in our original panel of normals (19.22%). We assessed the coverage of tumor-only HLA alleles in healthy samples using the panel of normal samples. The 334 healthy samples covered 53% of the HLA alleles expressed in the tumor samples. Analysis of a subset of ncMAPs represented by the 57 shared alleles (i.e., present in both healthy and tumor samples) revealed a substantial overlap in HLA-binding motifs between the panel of normal samples and other samples. This was demonstrated by the majority of identified ncMAPs being retained (7,513 of 8,601) and a comparable percentage of ncMAPs being detected in healthy samples through MS (36.46% with shared alleles versus 32% with all alleles; see Supplementary Fig. S7). To better understand the similarity between the HLA-binding motifs of the alleles represented in tumor-only samples and those represented in healthy samples, we generated a matrix of cosine distances of binding affinities and used t-SNE to reduce the dimensionality and visualize the data. Our results indicated a high level of similarity between the two, further supporting the notion that the 65 alleles in the panel of normal samples were representative of the tumor-only alleles (Supplementary Fig. S7).

However, the lack of ncMAP detection in the panel of normals does not confirm cancer selectivity owing to the sensitivity limitations of MS. Proper cancer selectivity assessment should be performed at the gene expression level in healthy tissues. Hence, we retrieved the parent gene expression values (in TPM) of the remaining ncMAPs from the Genotype-Tissue Expression project (GTEx v8; ref. 51). We first compared the gene expression levels of the following two groups: ncMAPs detected in the panel of normals by MS (blue), and remaining ncMAPs without detection in the panel of normals (red). Fig. 6B shows significantly higher gene expression for ncMAPs detected in healthy tissues (blue) than for those that were left undetected (red). Moreover, to ensure low toxicity levels in normal tissues, we filtered ncMAPs to retain those with parent genes expressed below 1 TPM and without evidence of protein expression in any healthy tissue except the testis (immune-privileged site; Fig. 6C). By applying this stringent filter, we identified 24 ncMAPs derived from genes not expressed or expressed only in trace amounts in healthy tissues. Of these, 17 were associated with proteins not detected in healthy tissues. Table 1 provides a summary of these 17 cancer-selective ncMAPs, which we suggest as promising targets for clinical applications (see Supplementary Table S3 for more details).

**Table 1.** List of cancer-selective noncanonical MHC-associated peptides.

ID	Peptide	Gene name	Mean expression in healthy tissues (TPM)	Number of healthy tissues with protein expression	Annotation
1	AFAPFPTQF	*CXorf49B*	0.01	0 of 56	Cancer selective
1	AFAPFPTQF	*CXorf49*	0.01	0 of 56	Cancer selective
1	AFAPFPTQF	*RP11–402P6.15*	0.10	0 of 56	Cancer selective
2	DYIHFVHHF	*RP11–325B23.2*	0.00	0 of 56	Cancer selective
3	EALSASQALYTR	*HIST1H4L*	0.04	43 of 56	
4	ELIKAFSK	*GNGT1*	0.05	1 of 56	
5	ESAGLFQVPR	*SUN3*	0.13	3 of 56	
6	EVEKILIQY	*KCNU1*	0.05	0 of 56	Cancer selective
7	EVPGAQGQQGPR	*CTAG2*	0.15	0 of 56	Cancer selective
7	EVPGAQGQQGPR	*CTAG1B*	0.03	0 of 56	Cancer selective
7	EVPGAQGQQGPR	*CTAG1A*	0.06	0 of 56	Cancer selective
8	FPVDVDHAVL	*CTAG2*	0.15	0 of 56	Cancer selective
8	FPVDVDHAVL	*CTAG1B*	0.03	0 of 56	Cancer selective
8	FPVDVDHAVL	*CTAG1A*	0.06	0 of 56	Cancer selective
9	ILSDNIRNL	*C1orf94*	0.14	0 of 56	Cancer selective
10	IPKDKSKNK	*C2orf83*	0.02	0 of 56	Cancer selective
11	KLLELIKAFSK	*GNGT1*	0.05	1 of 56	
12	KNNIYAFKI	*RP11–231I13.2*	0.01	0 of 56	Cancer selective
13	KTLHLTIVK	*C12orf50*	0.07	0 of 56	Cancer selective
14	KYLSRFRPK	*TRPC5*	0.08	0 of 56	Cancer selective
15	MVRSPEEGSLR	*TEX19*	0.13	0 of 56	Cancer selective
16	MVRSVSAAAR	*HIST1H2BB*	0.26	44 of 56	
17	MVRSVSAAARR	*HIST1H2BB*	0.26	44 of 56	
18	REEAPRGVRM	*CTAG2*	0.15	0 of 56	Cancer selective
18	REEAPRGVRM	*CTAG1B*	0.03	0 of 56	Cancer selective
18	REEAPRGVRM	*CTAG1A*	0.06	0 of 56	Cancer selective
19	SAGLFQVPR	*SUN3*	0.13	3 of 56	
20	SQVHKFFLL	*OR9Q1*	0.04	0 of 56	Cancer selective
21	SYGIYIYTY	*SLC15A5*	0.06	0 of 56	Cancer selective
22	TVSHQIIFY	*EXD1*	0.06	0 of 56	Cancer selective
23	VIQKVILVV	*MGAT4D*	0.03	0 of 56	Cancer selective
24	YYFILEHAKY	*SOX30*	0.29	0 of 56	Cancer selective

Note: The mean parent gene expression in TPM was derived from 29 healthy tissues from the GTEx v8 dataset. The number of healthy tissues with protein expression was obtained from the Human Protein Atlas v22.0.

## Discussion

The cartography of noncanonical antigen presentation revealed in our study arose from a harmonized large-scale analysis of immunopeptidomic data mapped to the human genome. Our innovations over the most recent trends in computational MS identified a diversity of peptides mapping to canonical and noncanonical translation products. We mapped deviations away from the reference proteome as mass shifts (PTMs) and applied a sequential approach to tackle the noncanonical immunopeptidome. Our proteogenomic pipeline allowed the identification of thousands of ncMAPs (8,601) derived from noncoding regions of protein-coding genes with an FDR of 1%. This was accomplished by analyzing a large collection of publicly available studies using COD-dipp, a highly modular large-scale pipeline that bypasses the challenge of multi-omics requirements and large MS databases when identifying ncMAPs.

Recent studies have suggested that the immunopeptidome is rich in PTMs (62), which can have profound effects on immune tolerance. T cells can discriminate between modified and nonmodified epitopes, which has been demonstrated in the case of ubiquitination (63), glycosylation (64), phosphorylation (1, 65). T-cell reactivity to PTMs is an effect of their central tolerance escape from the thymus (66). PTMs may also alter proteolytic activity, and consequently, peptide presentation by the MHC system (67). The open-search component sheds light on several PTMs implicated in immunogenicity (serine N-terminal acetylation, cysteinylation, and cysteine tri-oxidation) and could provide insights for future studies on PTM-based epitopes. For instance, tri-oxidation of cysteine has the potential to alter the immune response (55); however, its mechanism of interaction with HLA molecules and T cells is still in its infancy. Additionally, T cells can discriminate between cysteinylated and unmodified cysteine residues (56). Likewise, N-terminal serine acetylation is known for multifunctional regulation, acting as a protein degradation signal, inhibitor of endoplasmic reticulum (ER) translocation, and mediator of protein complex formation. Methionine sulfone (methionine dioxidation) has been found to occur *in vivo* in *Proteus mirabilis* (68), a Gram-negative bacterium present in malignant cancers (69), although it can result from the use of a strong oxidizing agent.

The validity of ncMAPs was rigorously tested using retention time correlation (experimental vs. theoretical), orthogonal second-round search, mass accuracy, PTM retention time shifts, HLA binding prediction, and PSM comparison with previously published results. Twenty-five percent of the identified ncMAPs accounted, on average, for 81.36% of intersections when compared with three other high-profile studies (6, 13, 16). In addition, COD-dipp revealed 6,433 new ncMAPs from protein-coding genes. Considering the high-quality and rigorous computational validation, the identification rate discrepancy is partly due to the performance of COD-dipp and the size of our dataset collection, making it the most exhaustive noncanonical library of MHC class I–associated peptides.

Our survey of the possible sources of ncMAPs revealed that 70.1% could be attributed to nORFs, IR, or frameshift mutations. We identified 597 ncMAPs downstream of known frameshift mutations in COSMIC, an understudied source of antigens in immunopeptidomic studies. Certainly, other biological processes not accounted for in this study could generate ncMAPs. For instance, mechanisms such as ribosomal slippage (11) and stop codon readthrough could explain some of the remaining ncMAPs (29.9%).

This study focuses on peptides from noncoding regions of the genome, referred to as noncanonical peptides. Unlike neoantigens, which derive from patient-specific mutations in cancer, these noncanonical peptides are not mutated and are present in both cancer and healthy individuals. Although their presence in healthy samples makes their tumor specificity less clear, noncanonical peptides tend to be more abundant in cancer cells than in healthy cells. Over two decades ago, Ishii and colleagues (70) purified an octamer noncanonical antigen (IPGLPLSL or pRL1a) associated with heat shock proteins (HSP) and validated their findings using MS. The isolated octamer noncanonical antigen pRL1a was derived from the 5′-untranslated region of the *AKT* gene in leukemia and induced tumor rejection. To the best of our knowledge, this was the first demonstration of a noncanonical antigen that confers immunity. Subsequent studies have shown that HSPs are beneficial for anticancer vaccines (71) because they bind canonical/noncanonical antigens with tumor rejection properties that end up being presented by MHC I and II molecules (72).

Numerous studies have suggested various possible candidates for cancer vaccines over the past two to three decades, and each has failed, at least partly, due to the issue of specificity. We used a conservative definition of cancer selectivity that follows three iterative steps. We searched for the identified ncMAPs over a panel of 334 normal MS samples and confirmed a fraction (32%) of noncancer–selective ncMAPs. The remaining fraction (5,843, 68%) contained both cancer-selective ncMAPs and noncancer–selective ncMAPs that were not detected by MS. We used the expression levels of the ncMAPs’ parent genes across 29 healthy tissues as a means of prioritization (6, 14, 16). ncMAPs whose parent genes were expressed in any normal tissue above a threshold of 1 TPM were not considered cancer selective. However, we caution that this definition excludes the consideration of 92% of protein-coding genes. We revealed 17 rigidly defined candidates as cancer-selective ncMAPs, originating from genes and proteins that were completely absent or available in trace amounts in healthy tissues. We hope that this offers a sufficiently stringent approach to reducing toxicity in clinical applications. We provide a complete breakdown of all detected ncMAPs in Supplementary Table S3. We report the parent gene and protein expression values across healthy tissue types from the GTEx cohort and Human Protein Atlas, respectively. Moreover, we report their cancer-selectivity status conditioned on a gene expression cutoff (1 TPM) and lack of protein expression in healthy tissues. This will allow the research community to make decisions regarding the peptides that should be retained or removed from their analyses. It is particularly important that we do not filter all peptides, as aberrant intron-retention and frame-shift mutations that are certainly cancer-specific may lie within these results and would not need this stringent filtering if found in subsequent studies.

Here, we provide a free and open-source informatics pipeline to study noncanonical peptides, along with a reservoir of potential targets that could be used in combination with T-cell therapies or cancer vaccines. We anticipate that this will help pave the way for future research on antigens from noncanonical sources and engage further oncology research on alternative sources of antigens.

We acknowledge that our study presents several limitations. First, our approach relies on a DDA MS, which is known for its dynamic range limitations. Thus, only the most abundant ncMAPs were identified. Moreover, owing to the technical limitations of MS, we require that our ncMAPs be at least 3 amino acids different from any known human protein. Thus, a substantial fraction could be eliminated, leading to underestimation of the noncanonical fraction. Second, because immunogenicity prediction is still in its infancy, the identified ncMAPs require further validation to qualify as tumor rejection–mediating antigens for clinical applications. Despite our efforts to identify cancer-selective targets, the toxicity of these peptides in healthy tissues requires further investigation.

## Supplementary Material

Supplementary figures Supplementary Figures S1 to S7.

Supplementary legends Legends for Supplementary tables, supplementary figures, and supplementary notes.

Supplementary notes Supplementary notes 1 and 2.

Supplementary Table 1 Datasets and samples considered in this study.

Supplementary Table 2 Open search peptide-spectrum matches (PSMs) information of serine acetylation, cysteinylation, carbamidomethylation, and cysteine trioxidation.

Supplementary Table 3 Annotation of the COD-dipp ncMAPs.

Supplementary Table 4 Comparison data of COD-dipp ncMAPs with 4 other studies.

Supplementary Table 5 Mapping of ncMAPs to COSMIC frameshift mutations.
