# Supplementary material for: The Immunopeptidome from a Genomic Perspective: Establishing the Noncanonical Landscape of MHC Class I–Associated Peptides
Source: Cancer Immunol Res. 2023 Mar 24;11(6):747–62. doi: 10.1158/2326-6066.CIR-22-0621 (PMC10236148; doi:10.1158/2326-6066.CIR-22-0621)
Supplement: Supplementary figures — S1 to S7. [file cir-22-0621_supplementary_figures_suppsf1-sf7.docx]

# Figure S1

**
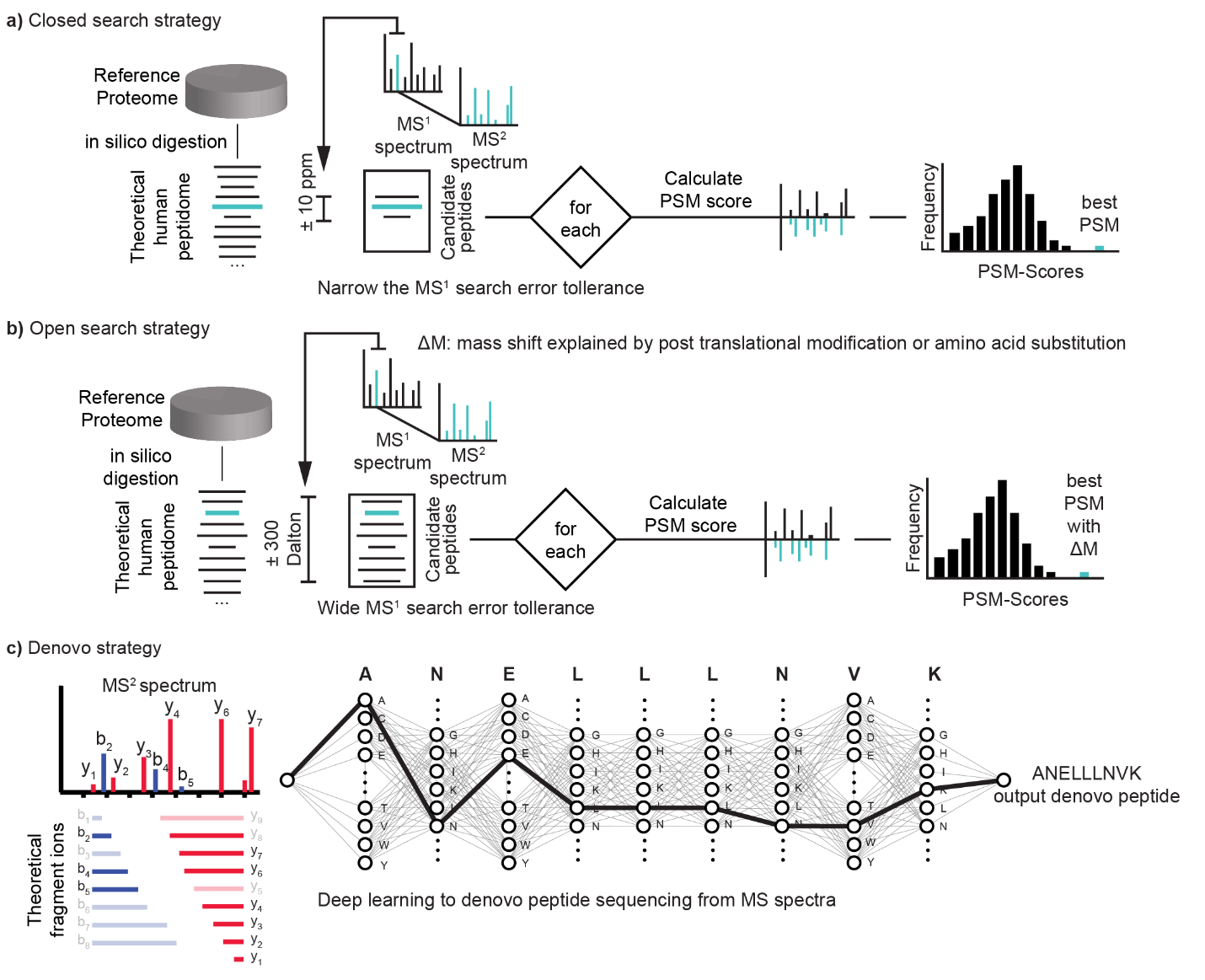
**

**Fig. S1: The mass spectrometry strategies used in this study. a)** A closed search approach (supervised approach) requires a reference protein sequence database that contains the expected proteins within the sample. These protein databases are in silico digested and peptides falling within a certain error tolerance are chosen as candidate peptide assignments. Each candidate peptide is then scored against the spectrum using an algorithm-specific methodology, and the highest scoring one is assigned as the sequence of the MS2 spectrum. **b)** Open search strategy (semi-supervised approach) widens the MS1 search error tolerance to identify peptides that would have been missed due to the mass shifts caused by mutations or post-translational modifications. **c)** *de novo* strategy (unsupervised approach) attempts to annotate spectra without a reference proteome by predicting a peptide sequence by directly reading the MS2 spectra.

# Figure S2


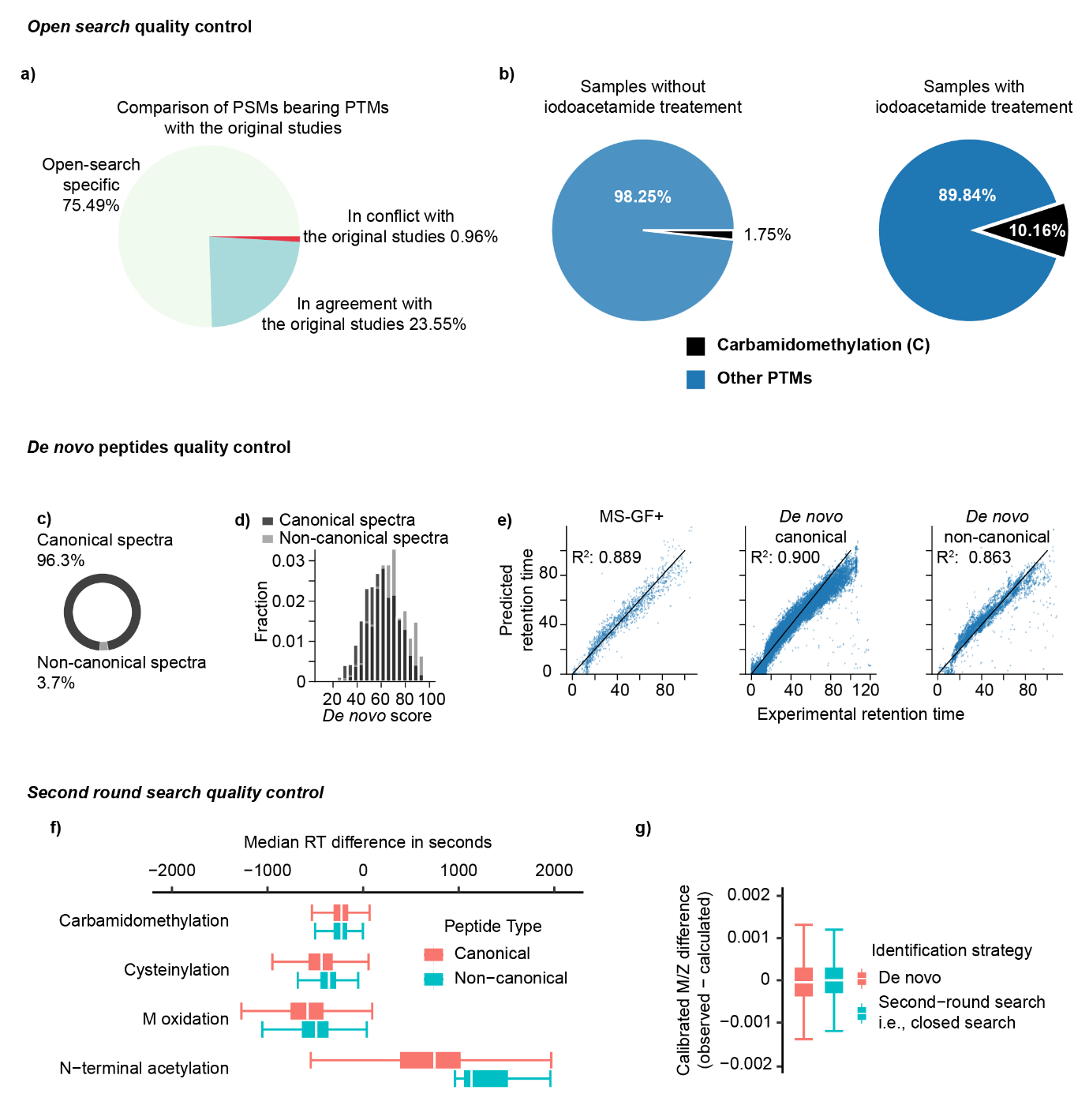


**Fig. S2:** **Additional quality control for the mass spectrometry peptide-spectrum matches (PSMs) of non-canonical and post-translationally modified MHC-associated peptides.** **a)** Comparison of PSMs identified by our open search with PSMs of the original studies. **b)** Percentage of carbamidomethylation within the subset of post-translationally modified peptide-spectrum matches for iodoacetamide-treated and untreated samples. **c)** *De novo* identified spectra from canonical (dark gray) and non-canonical (light gray) sources. **d)** *De novo* score distributions of canonical and non-canonical spectra. **e)** Correlation between predicted and experimental retention times for MS-GF+ and *de novo* peptides. **f)** Median retention time (RT) difference between peptides with and without a specific post-translational modification (PTM). Deviations between PTM-modified and unmodified canonical peptides are shown in red, and deviations between non-canonical peptides are shown in blue. Median RT refers to the retention time median value of multiple PSMs of the same peptides in a specific mass spectrometry run. The median RT difference refers to the difference between the median RT of the unmodified peptide and its modified counterpart. **g)** Boxplot of the mass differences between the observed mass over charge (M/Z) and the calculated M/Z of the non-canonical peptides identified using *de novo* (red) and second-round search (blue).

# Figure S3


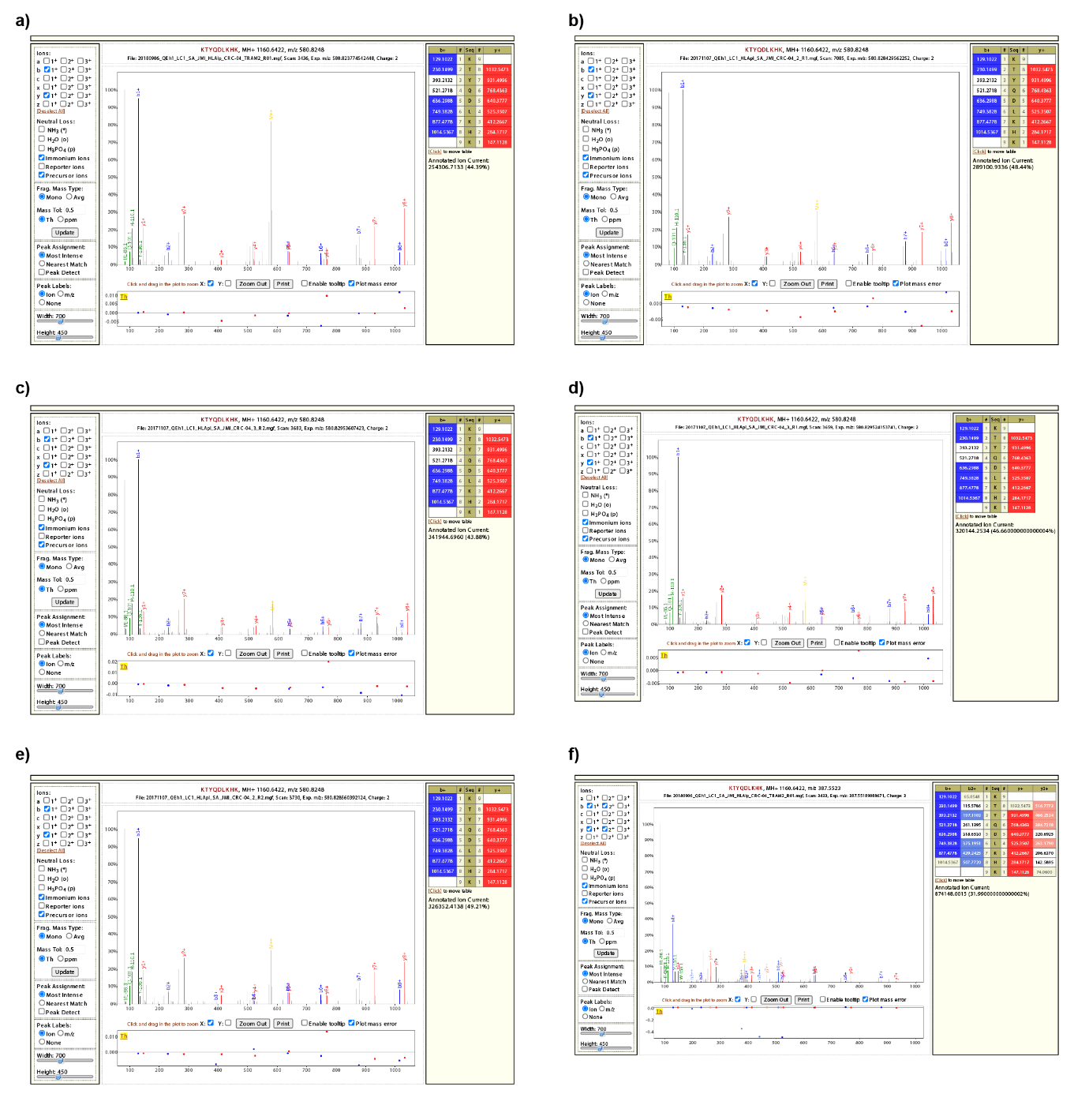


**Fig.S3: Spectra of the non-canonical MHC-associated peptide KTYQDLKHK from the PXD014017 dataset of a colorectal cancer patient (CRC-4).** **Panels a and e** show spectra from a replicate treated with trametinib. **Panels b, c, d, and f** show the spectra from four different replicates of the same patient (CRC-4) that were left untreated.

# Figure S4


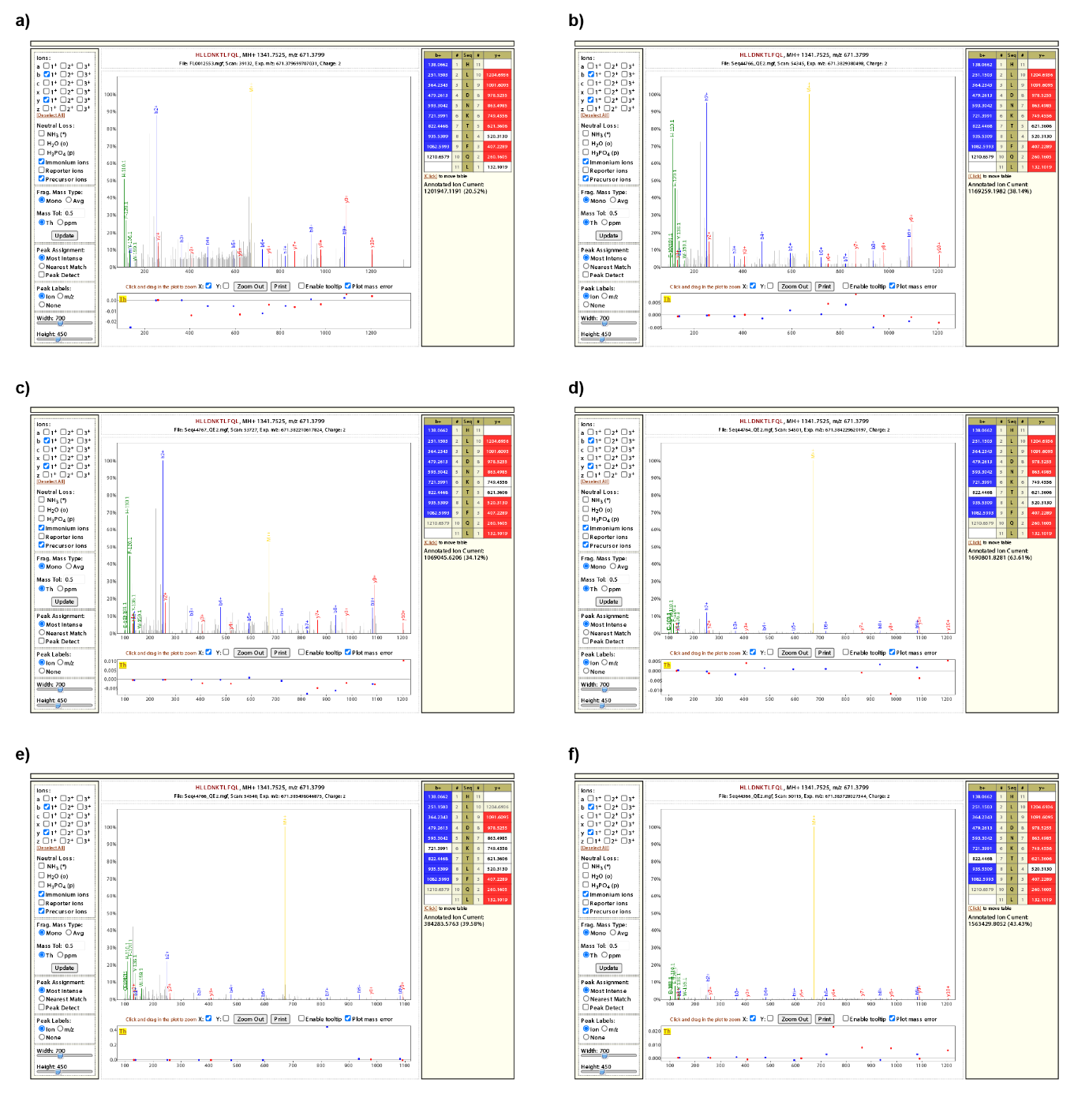


**Fig. S4:** **Spectra for the non-canonical MHC-associated peptide HLLDNKTLFQL from multiple datasets. Panel a** shows a spectrum from the PXD012083 dataset of an acute myeloid leukemia patient. **Panels b, c, and e** show spectra from the PXD003790 dataset of a brain glioblastoma cell line (T98G). **Panel d** shows a spectrum from the PXD003790 dataset of a brain glioblastoma cell line (U87). Panel (f) shows the spectrum from the PXD007596 dataset of a breast cancer cell line (MCF-7).

# Figure S5


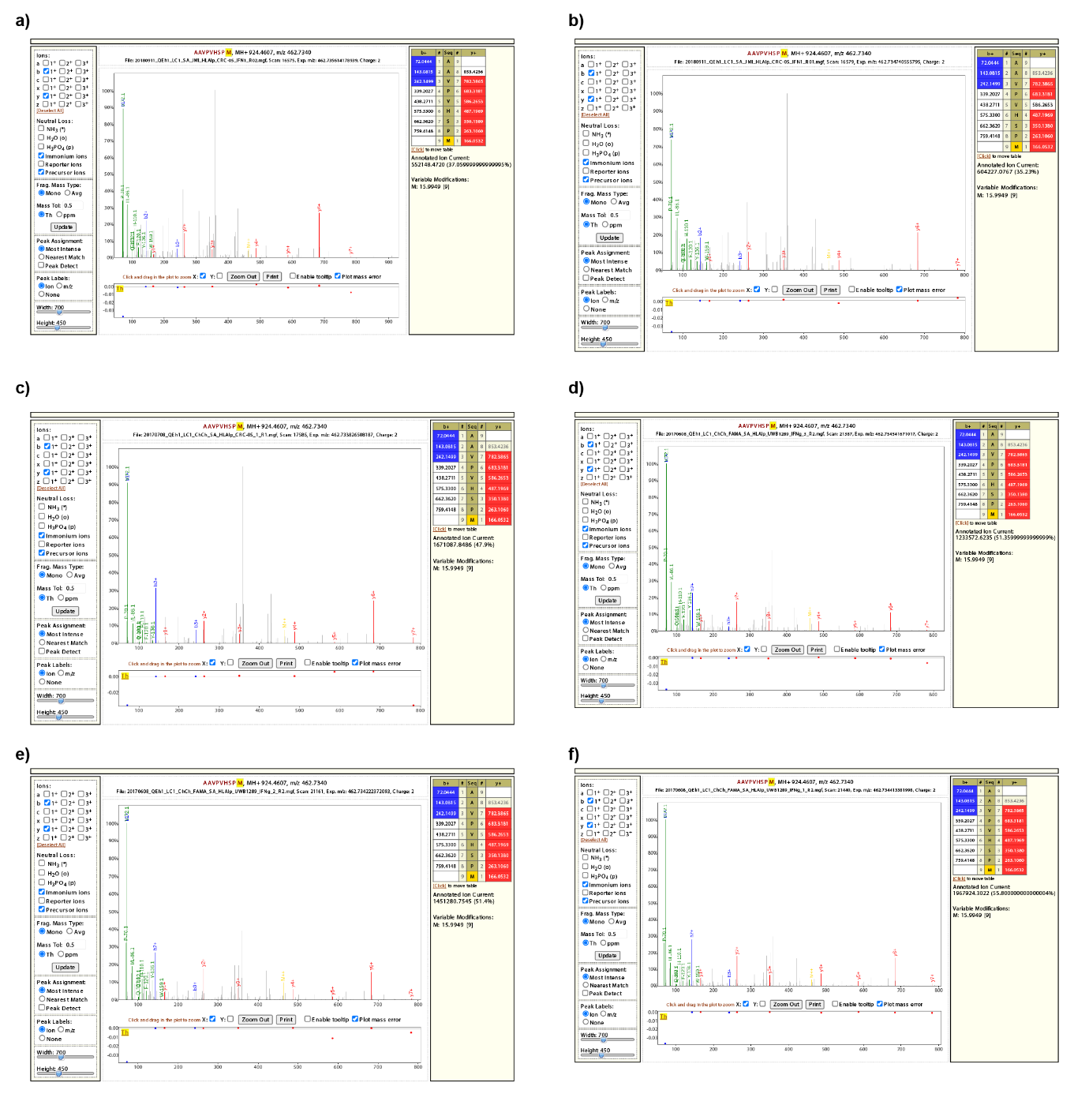


**Fig. S5: Spectra for the non-canonical MHC-associated peptide AAVPVHSPM(oxidation) from multiple datasets. Panels a and b** show the spectra from dataset PXD014017 of a colon cancer patient treated with IFN-γ. **Panel c** shows a spectrum from dataset PXD014017 of the same colon cancer patient who was left untreated. **Panels d, e, and f** show spectra from the PXD006939 dataset of an ovarian carcinoma cell line (UWB1289) for three different biological replicates.

# Figure S6

**
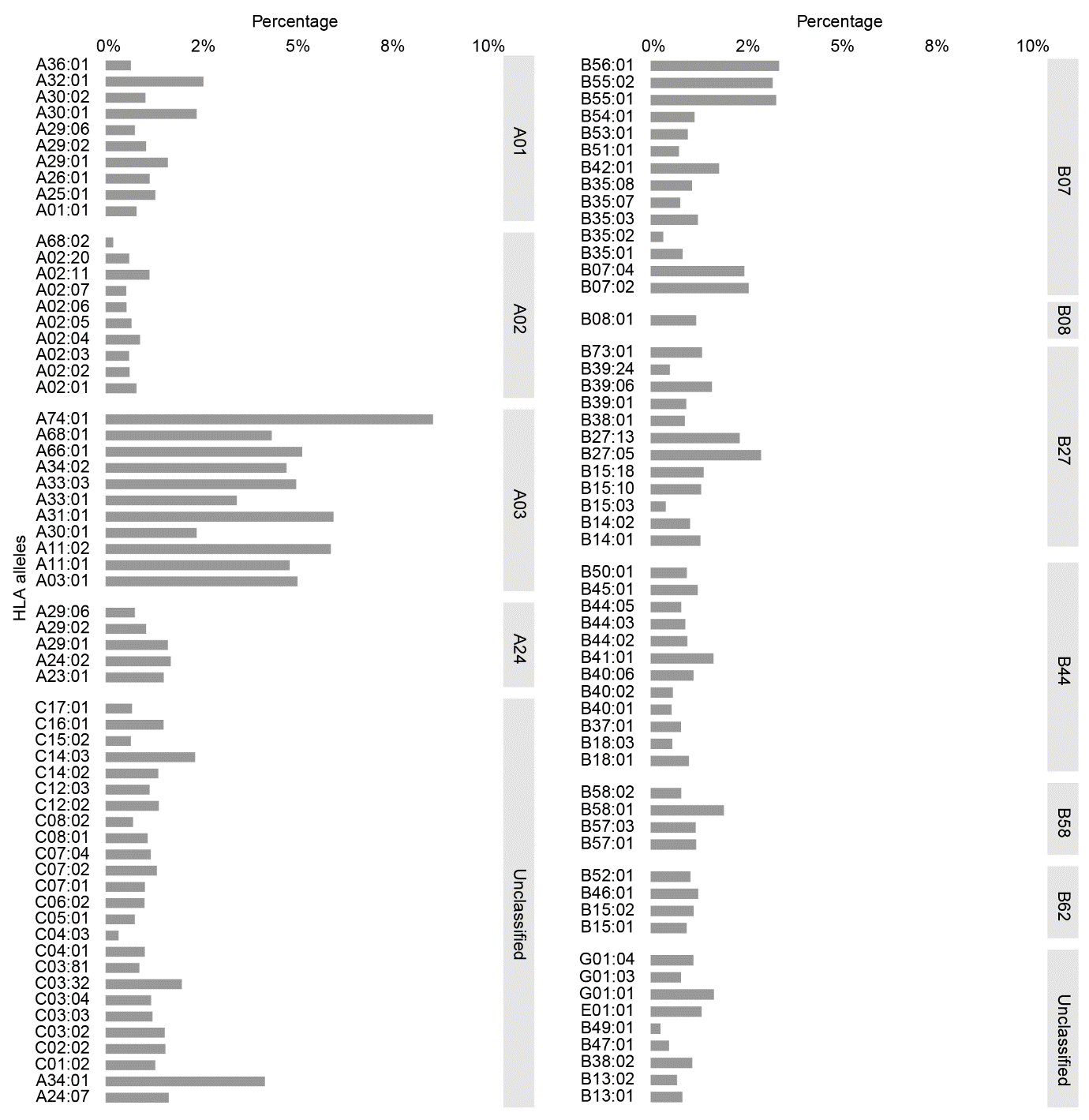
**

**Figure S6:** **HLA supertypes and non-canonical MHC-associated peptides (ncMAPs) expression.** The percentages of unique ncMAPs are shown for all 114 HLA alleles grouped into supertypes to reduce the complexity.

# Figure S7


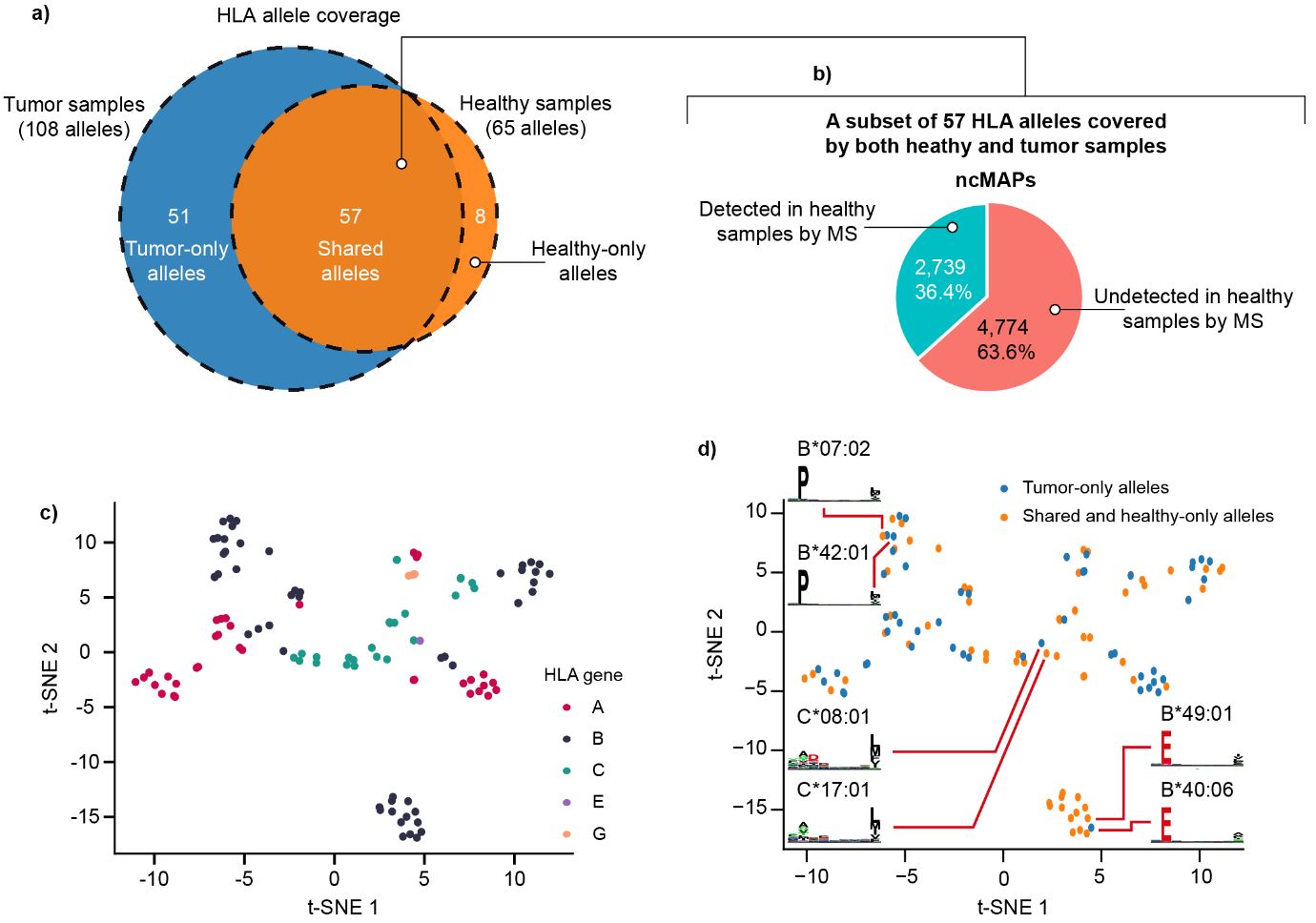


**Fig.S7:** **Comprehensiveness of the panel of normals in term of HLA-binding motifs.** **a)** HLA allele coverage intersection between tumor and healthy samples. **b)** Percentage of ncMAPs that are detected in healthy samples (red) versus those undetected in healthy samples (blue) for the subset of peptides presented by the shared allele *i.e.,* 57 HLA alleles common between tumor and healthy samples. **c)** HLA-binding landscape of all alleles colored by HLA gene type. **d)** HLA-binding landscape of all HLA alleles colored in blue for tumor-only alleles and in orange for shared or healthy-only alleles. Panels c and d show the similarity in HLA-binding motifs between all alleles in our dataset. As different HLA genes should present dissimilar binding motifs, panel c shows that different HLA genes map to distinct areas of the plot, supporting the idea that dissimilar HLA-binding motifs would appear in separate areas. Panel d shows a high similarity in the HLA-binding motifs between alleles covered by tumor-only samples (blue dots) and alleles covered by healthy samples (orange dots). In agreement with the findings of panel b, it is apparent that the 65 alleles in our panel of normals are representative of the tumor-only alleles in terms of HLA-binding motifs.
