# Supplementary material for: The Immunopeptidome from a Genomic Perspective: Establishing the Noncanonical Landscape of MHC Class I–Associated Peptides
Source: Cancer Immunol Res. 2023 Mar 24;11(6):747–62. doi: 10.1158/2326-6066.CIR-22-0621 (PMC10236148; doi:10.1158/2326-6066.CIR-22-0621)
Supplement: Supplementary legends — Legends for Supplementary tables, supplementary figures, and supplementary notes. [file cir-22-0621_supplementary_legends_suppsm1.docx]

**Supplementary Tables:**

1. Supplementary Table 1: Datasets and samples considered in this study.
2. Supplementary Table 2: Open search peptide-spectrum matches (PSMs) information of serine acetylation, cysteinylation, carbamidomethylation, and cysteine trioxidation.
3. Supplementary Table 3: Annotation of the COD-dipp ncMAPs.
4. Supplementary Table 4: Comparison data of COD-dipp ncMAPs with 4 other studies.
5. Supplementary Table 5: Mapping of ncMAPs to COSMIC frameshift mutations.

**Supplementary Figures**

1. Supplementary Figure 1: The mass spectrometry strategies used in this study.
2. Supplementary Figure 2: Additional quality control for the mass spectrometry peptide-spectrum matches (PSMs) of non-canonical and post-translationally modified MHC-associated peptides.
3. Supplementary Figure 3: Spectra of the non-canonical MHC-associated peptide KTYQDLKHK from the PXD014017 dataset of a colorectal cancer patient (CRC-4).
4. Supplementary Figure 4: Spectra for the non-canonical MHC-associated peptide HLLDNKTLFQL from multiple datasets.
5. Supplementary Figure 5: Spectra for the non-canonical MHC-associated peptide AAVPVHSPM(oxidation) from multiple datasets.
6. Supplementary Figure 6: HLA supertypes and non-canonical MHC-associated peptides (ncMAPs) expression.
7. Supplementary Figure 7: Comprehensiveness of the panel of normals in term of HLA-binding motifs.

**Supplementary Notes:**

1. Supplementary Note 1: List of keywords used to select datasets from PRIDE.
2. Supplementary Note 2: Correctness of the identified peptides.
