# Supplementary material for: The Immunopeptidome from a Genomic Perspective: Establishing the Noncanonical Landscape of MHC Class I–Associated Peptides
Source: Cancer Immunol Res. 2023 Mar 24;11(6):747–62. doi: 10.1158/2326-6066.CIR-22-0621 (PMC10236148; doi:10.1158/2326-6066.CIR-22-0621)
Supplement: Supplementary notes — 1 and 2. [file cir-22-0621_supplementary_notes_suppsm2.docx]

# Supplementary Notes

Table of content

[Supplementary Notes 1](#_Toc126753768)

[Note 1: Dataset selection 2](#_Toc126753769)

[Note 2: Correctness of the identified peptides 3](#_Toc126753770)

[Open-search quality control 3](#_Toc126753771)

[*De novo* quality control 4](#_Toc126753772)

[Second-round search quality control 4](#_Toc126753773)

# Note 1: Dataset selection

**List of keywords used for selecting datasets from PRIDE:** Immunoprecipitation, Immunopeptidome, Peptidomics, Affinity purification, Mhc, Peptidome, Hla, Immunopeptidomics, Mhc class i, Ip, Hla peptidome, Hla-b*27, Hla class ii, Neoantigens, Immunoinformatics, Hla-c, Mhc class 1 ligands, Proteogenomic cryptic mhc lc-msms maps, Mhc class i antigen presentation pathway, Mhc-i peptides, Mhc i, Immunopeptidome; hla; lc-ms/ms; netmhcpan; binding prediction, Mhc ii, Mhc-i peptide-loading complex, Mhc affinity prediction, Mhc-ii peptidomics, Mhc ligandome, Mhc i-associated peptides, Mhc-i, Mhc class ii, Antigen presentation/ mhc class ii/ immunopeptidome/ peptide editing/ polymorphism, Mhc-i peptidomics, Shotgun proteomics; immunoprecipitation; meiosis; conserved proteins; meioc; , Anti-hla immunopurification, Immunopeptidome; hla; lc-ms/ms; netmhcpan; binding prediction, Personalized immunotherapy, Immunoprecipation, Immunoprecipiation, Immunoaffinity purification, Immunoprepicipitations, Immunopurification, Antigen presentation/ mhc class ii/ immunopeptidome/ peptide editing/ polymorphism, Hla-ii, Hla peptides, Hla-e, Hla-b*51, Hla class i peptides, Ducaf; hla-drb1*03:01, Hla typing, Hla-g, 'Hla class ι ligandome; hla class ι peptide ligands; high ph reversed phase; strong cation exchange; pre-fractionation', Hla-b40, Hla binding motifs, Hla-dm, Hla-b27, Immunopeptidome; hla; lc-ms/ms; netmhcpan; binding prediction, Hla-b*58:01, Hla-b*40:02 peptidome, Hla-dr peptides, Hla-dr, Hla-a, Hla-b57, Hla class i, Hla-i, Hla-a2, Hla-b, Interferon gamma; proteomic analysis; hla class i; apm, Hla-i peptides, Hla-ligand, Hla-b*57:03, Hla-ligandomics, Hla-a*29:02, Hla-dr15, Hla-class i, Hla-restricted peptide

# Note 2: Correctness of the identified peptides

The most definite validation metric of correctness is shown by a high similarity between the MS/MS spectra of the endogenous and synthetic peptides, as well as the co-elution of the light and heavy peptide pairs. Considering the impossibility of performing such an analysis due to the reliance of this study on publicly available data. We have assessed the correctness of the identified peptides in a series of quality control experiments.

## Open-search quality control

**Validation 1:** We compared the identifications obtained with open search in this study with the identifications in the original studies at the peptide-spectrum match (PSM) level (*i.e.,* for each MS/MS spectrum). We successfully collected PSM information from 19 of the 25 analyzed datasets. The remaining 6 datasets presented some challenges. Three of these datasets (PXD004233, PXD008937, and PXD009531) reported PSM-level data but without FDR control, and three (PXD012083, PXD004746, and PXD010808) reported PSM in a format that prevented us from recovering the MS/MS scan numbers from the raw files. We compared the PSMs identified by our open search to those reported in the original studies and considered an agreement when the same mass spectrometry scan showed (I) an identical peptide sequence and (II) an identical mass shift introduced by the PTM. We found that 96.1% of the modified PSMs were identical in both sources (49,918 out of 51,945). To expand on the remaining 3.9% of the PSMs that were in conflict, we inspected the discrepancies and determined that they consisted of PTMs with monoisotopic masses close to those of some amino acids. For example, N-term glycidamide (87.03203 Da) can be misinterpreted as serine (87.0782 Da), carbamidomethyl (57.02146 Da) as glycine (57.02146 Da), N-term Propionamide (71.03711 Da) as alanine (71.03711 Da), phenethyl isothiocyanate (163.04557 Da) as tyrosine (163.1760 Da), N-term dicarbamidomethyl (114.04293 Da) as asparagine (114.04293 Da), and 4-hydroxynonenal (156.115030 Da) as arginine (156.10111 Da). In these cases, the open-search identified peptides were one amino acid shorter on the N-termini while bearing a PTM with a monoisotopic mass close to the missing amino acid. We believe that this conflict stems from an incomplete fragmentation pattern, in which the missing b1 and/or y(n) ions in the MS/MS spectrum leave the search engine with an equally fit decision to match it with the PTM- or non-PTM-bearing sequence.

To further validate our results and assess error rates, we employed a confirmatory procedure by evaluating the identification rate of cysteine carbamidomethylation in samples that were not treated with iodoacetamide. It is important to note that carbamidomethylation is a deliberate PTM introduced to cysteine residues through a reaction with iodoacetamide; thus, samples that have not undergone iodoacetamide treatment should not exhibit cysteine carbamidomethylation. To minimize false identifications, we applied stringent filters, including a global false discovery rate (FDR) and false localization rate (FLR) of 1%. This means that one would expect a 1% false identification rate, and approximately 1% for each group of PTMs. Our findings revealed that the samples lacking iodoacetamide treatment incorporated 1.75% of peptide-spectrum matches with cysteine carbamidomethylation, which we consider reasonable. It is important to note that this percentage represents a group FDR rather than a global FDR. As such, each PTM group would theoretically have a group FDR of approximately 1%, which would balance out to a global FDR value of 1% when considering all PTM groups together.

## *De novo* quality control

Our sequential *de novo* strategy showed that 96.3% of the MS spectra were canonical (*i.e.,* within known proteins) and a minority (3.7%) were non-canonical (*i.e.,* mapping to the 3-frame translation database).

**Validation 2:** We assessed the quality of the *de novo* sequences by examining their DeepNovoV2 scores. Canonical and non-canonical peptides had similar *de novo* score distributions, with a slight shift toward higher scores for non-canonical peptides.

**Validation 3:** We assessed the quality of the *de novo* sequences by examining the correlation between their experimental and theoretical liquid chromatography retention times. Canonical and non-canonical *de novo* sequences had a high correlation, with an R^2^ score of 0.9 for *de novo* canonical and 0.863 for *de novo* non-canonical peptides in a melanoma sample (mel-15 from PXD004894), and an overall *de novo* non-canonical R^2^ score of 0.88 among all samples.

## Second-round search quality control

**Validation 4:** The results of the second and third validations showed strong evidence that the *de novo* non-canonical peptides were of high quality (*i.e.,* correctly predicted complete peptide sequences). Even with this strong evidence, it is possible that chromatic behavior remains unchanged in certain instances where neighboring amino acids are in flipped positions, or that a 90% accuracy rate still leads to an uncertain false discovery rate percentage. Hence, we confirmed the identified 10,413 *de novo*-based ncMAPs by performing a second-round search for additional validation and controlling the FDR at 1%. The second-round search recovered 7,029 of the 10,413 *de novo*-based ncMAPs, with 76.52% (5,379) recovered from the same spectra (at least one spectrum per peptide). Overall, the second-round search identified 8,601 ncMAPs with a subset of 1,572 ladder sequences (subsequences) after cleavage of the 10,413 *de novo*-based ncMAPs by the search engine. As for post-translationally modified peptides, the second-round search recovered 51.85% of N-terminal acetylated peptides, 27.96% of peptides with cysteine carbamidomethylation, 74.75% of peptides with cysteinylation, and 71.02% of peptides with oxidized methionine from the same spectra (at least one spectrum per peptide). The low recovery of carbamidomethylation was mostly due to incorrect open-search assignments in iodoacetamide-untreated samples, considering that 81.01% was recovered by the second-round search in iodoacetamide-treated samples.

**Validation 5:** We confirmed that post-translationally modified peptides from the second-round search exhibited a shift in retention time that was consistent with that of their unmodified counterparts. Furthermore, we observed that for a specific PTM, there was a similar shift in retention time between non-canonical and canonical MHC-associated peptides. In each case, the modification caused the retention times of PTM-bearing non-canonical MHC-associated peptides to deviate in the same direction relative to the unmodified peptides. We found a high degree of agreement in retention time shifts between canonical and non-canonical peptides for three PTMs: carbamidomethylation, cysteinylation, and methionine oxidation. For N-terminal acetylation, the quantile ranges (Q1-Q3) were shifted between the two categories. However, it is important to note that the non-canonical category still fell within the standard range of the canonical category, which was mostly due to the low number of identified non-canonical N-terminal acetylated peptides with unmodified counterparts (9) compared to the large number in the canonical group (426).

**Validation 6:** We checked the mass difference between the observed and calculated masses (i.e., theoretical mass) of the peptide-spectrum matches (PSMs). We isolated the PSMs identified by the *de novo* strategy as well as those validated by second-round search. A similar distribution of mass differences between the *de novo* identified peptides (from -0.0014 to 0.0013 mass (M) / charge (Z)) and the second-round search validated from -0.0012 to 0.0012 M/Z) was observed.

**Validation 7:** We performed a comprehensive comparison between the PSMs obtained from our second-round search and those reported in the original studies. We hypothesized that if the non-canonical peptides were correctly identified, they would not have been recognized by the original studies that focused on detecting only canonical peptides originating from the proteome. Our analysis showed a remarkable correlation with our hypothesis, as 98.87% (9,495,747 of 9,508,165) of non-canonical PSMs were not detected in the original studies.
